# Range-based finishing enhances omega-3 fatty acids and forage-derived metabolites in bison meat across a seven-treatment comparison

**DOI:** 10.1186/s40104-026-01513-9

**Published:** 2026-11-05

**Authors:** Joseph V. Varre, Garrett Weldy, Lydia O’Sullivan, Amanda Blair, Robert Ward, Muhammad Ahsin, Ying Xi, Carter Kruse, Stephan van Vliet

**Affiliations:** 1 Department of Nutrition, Dietetics and Food Science, Utah State University, Logan, UT USA; 2 Department of Animal Science, South Dakota State University, Brookings, SD USA; 3 Department of Animal and Equine Science, Murray State University, Murray, KY USA; 4 Turner Institute of Ecoagriculture, Bozeman, MT USA

**Keywords:** Bison, Fatty acids, Finishing system, Metabolomics, Nutrient density, Range-finished

## Abstract

**Background:**

Finishing practices influence the biochemical composition of bison (*Bison bison*) meat, with potential implications for animal health and human nutrition. This study evaluated seven representative finishing systems spanning a range of extensive and intensive production systems, with additional contrasts in forage diversity, corn supplementation, and stocking density to determine their effects on the fatty acid and phytonutrient profiles of bison striploins (*M. longissimus lumborum*). Treatments included range-finished bison grazing either diverse native pastures (~200 plant species) or lower-diversity pastures (~90 plant species), with or without corn supplementation, as well as pen-finished bison managed under varying feed formulations and stocking densities, including a conventional total mixed ration (TMR) group. Fatty acids were analyzed by gas chromatography–flame ionization detection (GC-FID), and targeted metabolomic profiling of phytonutrient-derived metabolites was performed using liquid chromatography–tandem mass spectrometry (LC–MS/MS).

**Results:**

Range-finished bison exhibited higher concentrations of alpha-linolenic acid (ALA; C18:3n-3; *P* < 0.0001), docosapentaenoic acid (DPA; C22:5n-3; *P* < 0.0001), and docosahexaenoic acid (DHA; C22:6n-3; *P* < 0.0001), and lower n-6:n-3 ratios (2.8–3.6) than pen-finished bison (5.0–7.3; *P* < 0.0001). Metabolomic profiling revealed enrichment of hippuric acid, catechol, and shikimic acid (candidate biomarkers associated with forage intake and polyphenolic metabolism) in range-finished systems, whereas 3-(4-hydroxyphenyl)lactic acid, a potential grain-associated metabolite, was higher in pen-finished bison than in the unsupplemented range-finished systems (all *P* < 0.05).

**Conclusions:**

Range-finished systems enhanced omega-3 fatty acid and phytonutrient-related metabolite profiles, whereas the conventional TMR feedlot treatment yielded the lowest levels and the free-choice corn-and-hay pen system yielded an intermediate profile. Overall, the clearest nutritional contrast was between range-finished and pen-finished systems, whereas differences attributable to forage diversity or stocking density were comparatively modest. These findings indicate that finishing system strongly influences the biochemical composition of bison meat, although comparisons involving the conventional TMR feedlot treatment should be interpreted cautiously because that group originated from a separate breeding herd.

**Supplementary Information:**

The online version contains supplementary material available at https://doi.org/10.1186/s40104-026-01513-9.

## Background

The nutrient composition of red meat is increasingly recognized as a function of animal genetics and management systems [1]. Among these, finishing strategies, including diet type, forage diversity, and stocking density, play a potential role in determining the biochemical quality of meat by affecting lipid metabolism, ruminal biohydrogenation [2], and exposure to plant primary and secondary metabolites [3]. In ruminant species, forage-based diets typically enhance the concentrations of eicosapentaenoic acid (EPA), docosapentaenoic acid (DPA), docosahexaenoic acid (DHA), conjugated linoleic acid (CLA), and phytochemicals, while decreasing n-6:n-3 ratios relative to grain-based feeding systems [4, 5]. These compositional differences have potential implications for human health, as a higher dietary intake of n-3 polyunsaturated fatty acids (PUFAs) and lower n-6:n-3 ratios are associated with improved cardiometabolic and inflammatory profiles [6], and circulating omega-3 fatty acids in consumers can be increased through the consumption of ruminant-based meats [7].

The North American bison industry encompasses a continuum of production systems, ranging from extensive native rangeland grazing and finishing (i.e., grass-finishing) to confinement finishing on total mixed rations (TMR; i.e., grain-finishing) [8, 9]. This diversity reflects both ecological adaptation and economic strategy but also results in considerable variation in ruminant meat nutritional traits [10]. Previous studies on beef and lamb have found that the finishing environment can markedly alter the tissue fatty acid composition and metabolite signatures [11, 12]. Recent work on bison has similarly found that pasture finishing enhances long-chain n-3 fatty acids and phytonutrient antioxidants, whereas confinement systems promote higher oleic and n-6 fatty acids, mirroring the findings in beef [13]. However, there is limited integrated evidence linking diverse finishing environments (including variations in forage composition, feed supplementation, and stocking density) to fatty acid and phytochemical composition of bison meat.

Beyond lipids, emerging metabolomic evidence finds that meat from range-finished animals contains distinct signatures of plant-derived and microbial metabolites, such as hippuric acid, cinnamic acid, and p-cresol metabolites, which reflect the intake and transformation of phenolics in the rumen and host tissues [14]. These metabolites may serve as biomarkers of production authenticity and have been suggested to be functionally relevant to human nutrition through antioxidant or anti-inflammatory actions, although their direct impact on human health via meat consumption requires further investigation [15]. Nonetheless, various plant secondary metabolites (e.g., flavonoids, non-flavonoid polyphenols, and simple phenols) have been found to benefit animal health and welfare due to their potential anti-inflammatory [16], anti-parasitic [17], and antioxidant effects [18].

The current study builds upon the recent work of O’Sullivan et al. [8], who comprehensively characterized the carcass and sensory attributes of bison heifers finished under the different management systems studied here. While that study focused on proximate composition and palatability, the present work extends those findings by examining the underlying biochemical and nutritional traits of meat derived from the same finishing treatments. Therefore, the objective of this study was to evaluate the impact of seven representative finishing systems, ranging from range-finishing to high-density confinement feeding, on the fatty acid composition and metabolomic profile of bison striploins. We hypothesized that bison finished under range conditions would exhibit lower n-6:n-3 ratios and higher concentrations of forage-associated phytochemical metabolites than those finished under pen-based TMR systems, and that corn supplementation on pasture would yield an intermediate profile. By linking the production environment to quantifiable biochemical outcomes, this study aimed to provide mechanistic insights into how ecological management and nutrient density are connected within bison production systems.

## Materials and methods

### Animals, diets, and sample collection

**Fig. 1:**
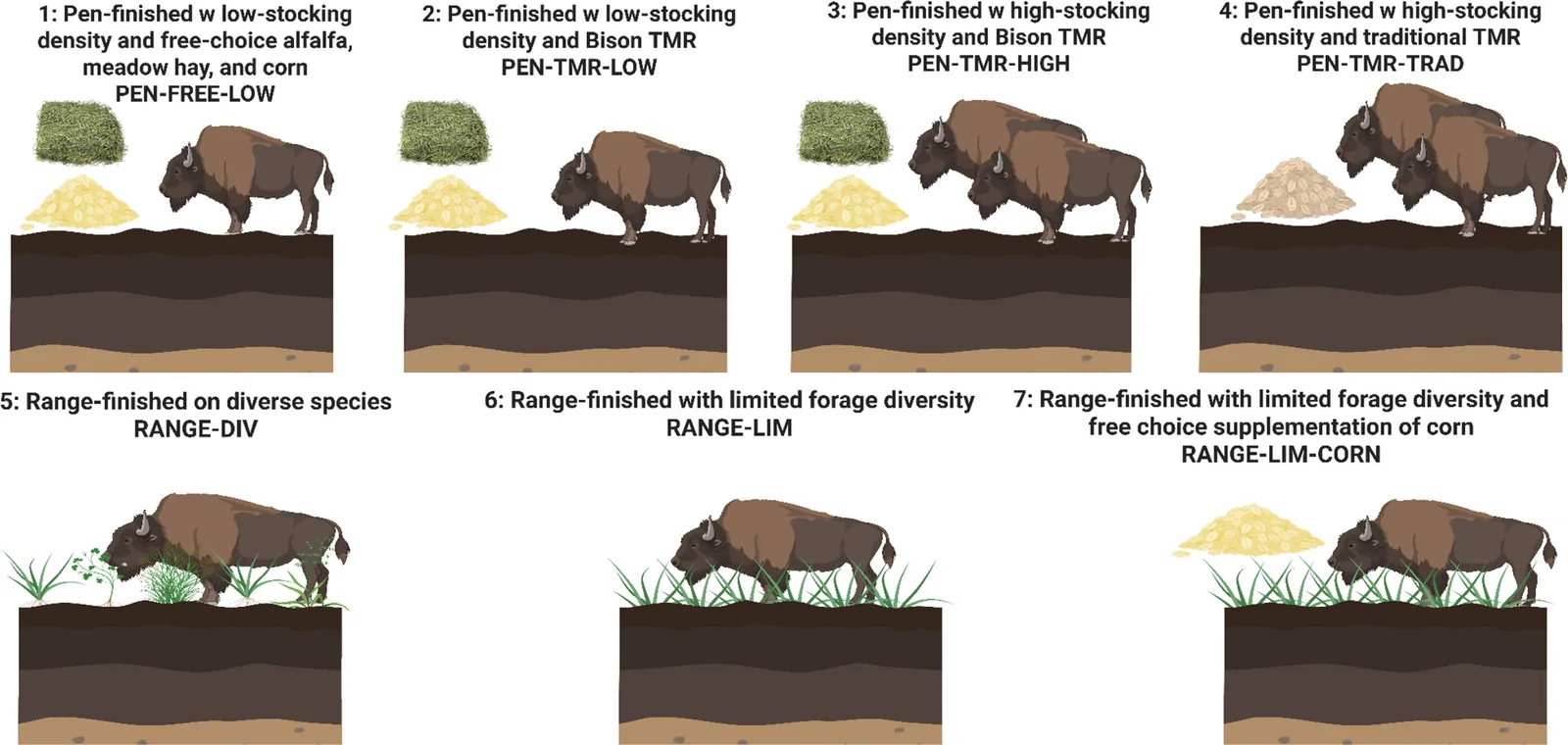
Overview of the bison finishing systems and study design. Bison heifers were finished under seven production systems differing in diet composition, forage diversity, pasture versus pen-based management, and stocking density. Pen-based systems included free-choice hay/corn feeding and total mixed ration (TMR) systems under low- and high-density conditions, including a traditional feedlot-style TMR system. Range-based systems included a botanically diverse native rangeland, a lower-diversity meadow pasture, and a lower-diversity pasture supplemented with corn. Following the finishing period, striploin samples (*M. longissimus lumborum*) were collected for fatty acid and targeted phytonutrient metabolomic analyses. Created with BioRender

Bison (*Bison bison*) heifers born in spring 2021 were studied in seven finishing systems designed to represent a range of management practices used in the North American bison industry. These systems varied primarily in diet composition and finishing environment, with additional contrasts in forage diversity and stocking density, to evaluate their effects on the fatty acid and phytonutrient profiles of bison meat (Fig. 1). Treatments 1–4 represented confinement-finishing environments: Pen-FREE-LOW (Treatment 1; *n* = 45) with free-choice access to alfalfa hay, meadow hay, and corn at low stocking density (55 m^2^ per animal); Pen-TMR-LOW (Treatment 2; *n* = 43), a bison total mixed ration (TMR) prepared from corn and oats mixed with ground alfalfa hay, ground grass hay, and added water, offered at low stocking density; Pen-TMR-HIGH (Treatment 3; *n* = 44), the same TMR offered at high stocking density (27 m^2^ per animal); and Pen-TMR-TRAD (Treatment 4; *n* = 45), a commercial high-density feedlot system typical of conventional bison production. Feed ingredients were analyzed by wet chemistry and adjusted during the trial in treatments 1–3 as needed to meet finishing requirements (Table 1).

**Table 1:** Standard feed analysis of rations used in the bison heifer finishing trial (dry matter basis)

Treatment	Feed type/description	DM, %	CP, %	NDF, %	ADF, %	TDN, %	NEm, Mcal/kg	RFV
1a	Pen-FREE-LOW – Whole corn	85.6	8.6	8.3	2.7	86.6	2.14	—
1b	Pen-FREE-LOW – Grass hay	90.1	10.4	67.1	42.0	55.2	1.15	78
1c	Pen-FREE-LOW – Alfalfa hay	82.3	20.2	53.5	42.2	53.4	1.08	97
2 & 3	Pen-TMR-LOW/Pen-TMR-HIGH (bison total mixed ration)	73.0	12.6	25.1	16.1	76.9	1.85	—
4	Pen-TMR-TRAD (commercial high-density feedlot ration)	—	—	—	—	—	—	—
5	Range-DIV (diverse native rangeland forage)	84.1	4.5	70.0	44.1	53.4	1.08	72
6	Range-LIM (low-diversity rangeland forage)	70.5	4.4	66.3	44.6	52.5	1.06	76
7	Range-LIM-CORN (low-diversity meadow forage)	65.4	6.8	65.6	43.2	54.3	1.12	78

Feed composition values are presented on a dry-matter basis. Samples were collected on 22 November 2023 and analyzed on 2 December 2023. Abbreviations include: *DM* Dry matter, *CP* Crude protein, *NDF* Neutral detergent fiber, *ADF* Acid detergent fiber, *TDN* Total digestible nutrients, *NEm* Net energy for maintenance, *RFV* Relative feed value. RFV values apply exclusively to forage components (Treatments 1b, 1c, 5, 6, and 7) and were not determined for total mixed rations (TMR) or corn samples. A representative feed sample was collected for Treatment 4 (Pen-TMR-TRAD), but proximate composition values were not available at the time of analysis

Treatments 5–7 represented extensive pasture-finishing environments: Range-DIV (Treatment 5; *n* = 44) grazing native upland rangeland characterized by high botanical diversity (an estimated 200 + plant species) within a mixed-grass prairie containing grasses, forbs, and sedges; Range-LIM (Treatment 6; *n* = 44) grazing lowland meadow pasture with lower forage diversity (90 + plant species) dominated by sedge and grass species; and Range-LIM-CORN (Treatment 7; *n* = 43), a similar low-diversity pasture with free-choice access to whole-kernel corn. Animals in these treatments were managed at very low stocking densities to optimize forage utilization and prevent overgrazing. Water was provided ad libitum. Botanical diversity of the rangeland pastures (high vs. low) was visually assessed and represents the observations and experience of the staff at McGinley Ranch regarding the abundance of plant species.

All animals in Treatments 1–3 and 5–7 originated from the same breeding herd and were managed under low-stress handling protocols throughout the trial period at McGinley Ranch. Treatment 4 animals originated from a separate breeding herd and were finished at a commercial feedlot in South Dakota, USA, using a grain-based TMR. All pens (treatments 1–3) at McGinley Ranch were open-air, and feed and water availability were monitored daily to maintain ad libitum intake.

Prior to assignment, the bison were vaccinated in February 2022 (ViraShield 6 + Somnus, Mycoplasma, Alpha7/MB-1, Cydectin injection) and October 2022 (Safe-Guard drench, Nuplura, Mycoplasma). The heifer calves remained with mother cows until fall 2022 and were then separated from the breeding herd and maintained separately on pasture until treatment allocation. The animals were visually assessed daily for health and behavior across all systems throughout the finishing period. Finishing commenced in May 2023 and continued for approximately seven months until harvest in December 2023–January 2024.

At the conclusion of the finishing period, the bison were transported to a commercial slaughter facility inspected by the United States Department of Agriculture (USDA). The animals were stunned and dispatched by rapid exsanguination. Transportation distances ranged from approximately 402 km for McGinley Ranch animals (Treatments 1–3 and 5–7) to animals finished under the traditional feedlot system (Treatment 4), which were all slaughtered in the same facility. Live weight and hot carcass weight (HCW) were recorded before and after slaughter, respectively. After a 24-h chilling period, carcasses were ribbed between the 12^th^ and 13^th^ ribs, and ribeye area, backfat thickness, marbling score, and kidney fat percentage were determined by trained personnel according to the USDA beef grading guidelines reported by O’Sullivan et al. [8]. Harvest was scheduled on a fixed-age basis rather than on an individual-animal appraisal, and no numeric commercial finish specification, such as a target subcutaneous fat depth or body condition score, was applied by the cooperating ranch or processor. Heifers in the treatments that were offered grain (Range-LIM-CORN, Pen-FREE-LOW, Pen-TMR-LOW, and Pen-TMR-HIGH) were harvested at approximately 31 months of age, whereas heifers in the two grass-only range treatments (Range-DIV and Range-LIM) were left an additional month on pasture and harvested at approximately 32 months of age [8, 19]. The degree of finish actually reached under each system is therefore best described by the carcass measurements recorded on these same carcasses [8, 19]. Backfat thickness averaged 0.39 cm in Range-LIM and 0.47 cm in Range-DIV, 1.24 cm in Range-LIM-CORN, and 1.69 to 2.23 cm across the pen-finished systems. Marbling scores were 150 to 160 in the range-finished systems and 221 to 262 in the pen-finished systems (100 = practically devoid, 200 = traces). The traditional feedlot comparator (Pen-TMR-TRAD) originated from a separate herd and is not included in those carcass data.

The *M. longissimus lumborum* (striploin) was removed from the left side of a subsample of carcasses from each treatment (*n* = 30 carcasses closest to the average HCW per treatment), vacuum-packaged, and transported under refrigeration to the South Dakota State University Meat Laboratory for further fabrication and analysis. Upon arrival, the striploins were trimmed of external fat and fabricated into 2.54-cm steaks. One steak from each of 16 carcasses per treatment (*n* = 16), selected from carcasses closest to the average HCW, was aged for 14 d postmortem at 4 °C, vacuum-sealed, frozen (−20 °C), and sent to the Center for Human Nutrition at Utah State University for fatty acid analysis. Targeted metabolomic profiling was performed on a separate analytical subset of 108 steaks across the seven treatments (*n* = 13–18 per treatment). All samples were stored and handled under identical conditions to minimize the variability associated with postmortem storage or temperature fluctuations. For compositional assays, the steaks were thawed at 4 °C for 24 h, coarsely pulverized while frozen in liquid nitrogen, and then finely ground using an IKA A 11 basic Analytical Mill (2900000; IKA-Werke GmbH & Co. KG, Staufen, Germany), a batch analytical mill that does not use a grinding plate or sieve. The ground meat was manually mixed to ensure homogeneity and stored at −80 °C until fatty acid methyl ester (FAME) preparation and targeted metabolomic profiling. The mill was thoroughly cleaned between samples to prevent cross-contamination.

Representative feed and forage samples were collected during the finishing period for fatty acid and phytonutrient analyses. For Pen-FREE-LOW, separate samples of alfalfa hay, meadow hay, and whole corn were collected. Because these ingredients were offered free-choice, the Pen-FREE-LOW values shown in Table 2 were calculated as a dry-matter-weighted treatment-level composite of the offered ingredients rather than as a single consumed ration profile; oats recorded in the feed log were included within the grain fraction because a separate oats fatty acid sample was not collected for analysis. Pen-TMR-LOW and Pen-TMR-HIGH received the same bison TMR formulation (corn and oats mixed with ground alfalfa hay, ground grass hay, and added water), and a representative TMR sample was therefore used for both treatments. Representative samples were also collected from the traditional feedlot TMR and from the forages associated with the Range-DIV and Range-LIM treatments. For Range-LIM-CORN, Table 2 values were calculated as a treatment-level composite of low-diversity meadow forage plus the sampled corn supplement. Feed and forage samples were freeze-dried (Labconco FreeZone freeze dryer, Labconco Corporation, Kansas City, MO, USA; standard conditions: condenser temperature of approximately −50 °C and a chamber vacuum of ~0.1 mbar until fully dry), finely ground to a uniform powder using an IKA A 11 basic Analytical Mill (2900000; IKA-Werke GmbH & Co. KG, Staufen, Germany), which does not use a grinding plate or sieve, and stored at −80 °C until fatty acid and targeted metabolomic analyses.

**Table 2:** Feed fatty acid composition (data shown as % fatty acids)

Fatty acid	Pen-FREE-LOW^1^	Pen-TMR-LOW^1^	Pen-TMR-HIGH^1^	Pen-TMR-TRAD^1^	Range-DIV^1^	Range-LIM^1^	Range-LIM-CORN^1^
Myristic	0.25	0.19	0.19	0.14	2.23	1.72	0.46
Palmitic	16.44	16.60	16.60	14.49	21.87	23.02	16.36
Palmitoleic acid (*trans*)	0.44	0.26	0.26	0.12	0.96	1.00	0.39
Palmitoleic acid (*cis*)	0.13	0.16	0.16	0.15	0.19	0.27	0.15
Stearic	2.34	2.17	2.17	3.21	4.39	2.98	2.04
Elaidic acid	0.06	0.07	0.07	0.09	0.13	0.09	0.07
Oleic acid	22.47	22.84	22.84	23.96	12.46	6.64	21.89
Linoleic acid	49.70	51.39	51.39	52.27	29.98	22.46	44.04
Arachidic acid	0.61	0.56	0.56	0.48	3.01	1.64	0.77
Gamma linolenic acid	0.08	0.06	0.06	0.04	0.21	0.32	0.10
Alpha-linolenic acid	6.84	5.04	5.04	4.53	22.13	37.95	13.10
Behenic acid	0.65	0.66	0.66	0.52	2.44	1.92	0.63

^1^Treatments: Pen-FREE-LOW = free-choice alfalfa/hay/corn, low stocking density; Pen-TMR-LOW = bison total mixed ration (TMR), low density; Pen-TMR-HIGH = bison TMR, high density; Pen-TMR-TRAD = traditional TMR, high density (feedlot); Range-DIV = diverse native range; Range-LIM = low-diversity range; Range-LIM-CORN = low-diversity range + corn. Pen-FREE-LOW is shown as a single treatment column for manuscript presentation; values represent a dry-matter-weighted treatment-level composite of the offered alfalfa hay, grass hay, and grain fraction rather than a single consumed ration profile. Range-LIM-CORN values represent a treatment-level composite of low-diversity meadow forage plus the corn supplement. Pen-TMR-LOW and Pen-TMR-HIGH received the same TMR formulation, so values are identical because stocking density, not diet composition, was the experimental variable

### Fatty acid analysis

The fatty acid composition of *M. longissimus lumborum* and feed samples was determined by gas chromatography following lipid extraction and methylation into fatty acid methyl esters (FAMEs) according to the one-step method described by O’Fallon et al. [20]. Approximately 250 mg of homogenized muscle or ground feed/forage tissue was weighed into 16 mm × 125 mm screw-cap tubes, and 350 µL of 10 mol/L KOH in methanol and 3,150 µL of methanol were added, along with an internal standard (C13:0; Nu-Chek Prep, Elysian, MN, USA). The samples were vortexed and incubated in a water bath at 55 °C for 1.5 h with intermittent agitation every 20 min. Following saponification, 290 µL of 12 mol/L H₂SO₄ in methanol was added to induce methylation, and the samples were reincubated at 55 °C for an additional 1.5 h. After cooling to room temperature, 2 mL of hexane containing 0.01% butylated hydroxytoluene (BHT) and 1 mL of deionized water were added to the mixture. The tubes were vortexed for 30 s and centrifuged at 1,000 × *g* for 5 min to separate the organic phase. The upper hexane layer containing the FAMEs was transferred to autosampler vials for gas chromatographic analysis. FAMEs were quantified using a Shimadzu GC-2010 Plus Gas Chromatograph (Shimadzu Scientific Instruments, Columbia, MD, USA) equipped with a flame ionization detector and a 100 m × 0.25 mm internal diameter, 0.25 µm film-thickness SP-2560 capillary column (Supelco, Bellefonte, PA, USA). The injector and detector temperatures were maintained at 250 and 260 °C, respectively. The oven temperature program was as follows: initial 140 °C (hold 5 min), ramped at 5 °C/min to 240 °C, and held for 20 min. Helium served as the carrier gas at a constant flow rate of 1 mL/min. Extracted FAMEs were analyzed by gas chromatography with flame ionization detection, and concentrations were expressed as percentages of total identified fatty acids. Individual fatty acids were identified by comparing retention times with a 37-component FAME standard mix (Supelco 47885-U) and expressed as a percentage of the total identified fatty acids. Fatty acids were grouped by saturation class as saturated (SFA), monounsaturated (MUFA), or polyunsaturated (PUFA), and by family (n-3 and n-6 fatty acids). The n-6:n-3 ratio was calculated as the sum of n-6 divided by the sum of n-3 fatty acids. All chromatograms were processed using the Shimadzu LabSolutions software, and the peaks were manually inspected for integration accuracy. Fatty acid data were reported on a normalized basis (% of total FAMEs) for all the subsequent statistical analyses.

### Phytonutrient analysis

Targeted metabolomic profiling encompassed the compounds reported in Table S2 for *M. longissimus lumborum* and feed/forage samples. One hundred milligrams of fresh meat tissue and 30 mg of freeze-dried plant tissue were weighed into 2-mL tubes. A 5-mm stainless steel bead and 1,250 µL of pre-cooled (−20 °C) internal standard-spiked extraction solvent containing methyl tert-butyl ether (MTBE) and methanol (2:1, v/v) were added to each sample. Isotopically labeled internal standards included 4-hydroxybenzoic acid-^13^C_6_, apigenin-d_5_, benzoic acid-d_5_, genistein-d_5_, resveratrol-^13^C_6_, phenol-^13^C_6_, and *trans*-cinnamic acid-d_7_. Samples were lysed using a bead-beater set to 25 oscillations per second (TissueLyser II, QIAGEN, Hilden, Germany) for 2 min. Samples were then mixed with 750 µL of water, vortexed, and centrifuged at 16,000 × *g* for 10 min at 4 °C to induce phase separation. The MTBE layer was removed, and the lower phase was collected in a new set of tubes. Thereafter, 500 µL of methanol were added, and samples were stored at −80 °C overnight to facilitate protein precipitation. The next day, samples were centrifuged at 16,000 × *g* for 10 min at 4 °C and passed through a 0.22-µm polytetrafluoroethylene filter. Finally, samples were stored at −20 °C for 4 h, followed by a final centrifugation at 16,000 × *g* for 10 min at 4 °C, after which they were transferred to autosampler vials and stored at −80 °C until injection. For plant samples, the remaining pellet was subsequently washed with 1,000 µL of methanol, and 1,250 µL of internal standard-spiked 2 mol/L NaOH (same internal standards described above) was added for alkaline hydrolysis for 16 h in a water bath at 30 °C. The hydrolyzed plant extract containing bound phenolics was then acidified to pH 3 with concentrated formic acid and sonicated for 10 min at room temperature. Samples were then centrifuged at 16,000 × *g* for 10 min at 4 °C, filtered through a 0.22-µm membrane, and subjected to solid-phase extraction (SPE) using a Strata-X-Pro 96-well plate. During SPE, samples were washed with 1,250 µL of water in 0.1% formic acid and eluted with 1,250 µL of methanol in 0.1% formic acid. Compounds were separated on a Waters ACQUITY ultra-performance liquid chromatography (UPLC) system equipped with a 1.7 µm High Strength Silica (HSS) T3 column (100 mm × 2.1 mm; Waters Corporation, Milford, MA, USA) and coupled to a SCIEX QTRAP 5500 mass spectrometer (SCIEX, Framingham, MA, USA), with the column maintained at 30 °C. The mobile phases consisted of (A) water with 0.1% formic acid and (B) acetonitrile with 0.1% formic acid. The gradient elution program was as follows: 5% B for 2.1 min at 0.2 mL/min, ramping to 95% B over 14 min at a flow rate of 0.46 mL/min, followed by re-equilibration to 5% B for 4 min at 0.175 mL/min to maintain stable system pressure. All analytes were initially prepared as stock solutions ranging from 1 to 10 mmol/L in appropriate solvents based on solubility. Multiple reaction monitoring (MRM) methods were developed by direct infusion of each standard compound (1–5 μmol/L) using a 1-mL syringe at a flow rate of 5–20 μL/min to optimize Q1 and Q3 transitions, collision energy (CE), collision cell exit potential (CXP), and Q0 dissociation (Q0D), and were categorized based on ionization efficiency in positive and negative mode. The ion source conditions for both positive and negative assays were optimized separately using 15 representative phytonutrient standards for each mode. For negative mode, the optimized parameters were source temperature 550 °C, spray voltage −5,500 V, curtain gas at 40 psi, collision gas (CAD) set to 12, and ion source gases 1 and 2 at 70 psi. For positive mode, the spray voltage was set to +5,000 V. The MRM cycle time was set to 1,000 ms, with dwell times ranging from 3–40 ms depending on the number of transitions monitored.

### Quality control and data analysis

Quality control (QC) measures and calibration procedures were implemented in this study as per best practice in metabolomics studies. Specifically, after every 15 sample injections, a double blank (100% methanol) and a blank spiked only with isotopically labeled internal standards were injected as QC samples. All samples were processed in analytical batches of 24. Samples were randomized prior to instrumental analysis to minimize the batch effects and analytical bias. Data acquisition and processing were performed using the SCIEX OS software version 3.0 (SCIEX, Framingham, MA, USA). Peak integration was performed using the MQ4 algorithm with Gaussian smoothing set to a factor of 3. Compound identification was confirmed based on the presence of both quantifier and qualifier ion transitions and ion ratio consistency. A signal-to-noise (S/N) threshold of 10 was applied, and a noise percentage filter of 80% was used to ensure robust peak detection. Quantitative calibration curves were validated with a minimum regression coefficient (*R*^2^) of 0.90, in accordance with the standard metabolomics quality assurance guidelines [21].

### Statistical analysis

Fatty acid data were analyzed using GraphPad Prism version 10.1.1 (GraphPad Software, San Diego, CA, USA), whereas meat phytonutrient data were analyzed using MetaboAnalyst 6.0. Differences among the seven finishing systems were determined using one-way analysis of variance (ANOVA), with the finishing system as a fixed effect. Treatments 1–3 and 5–7 formed the completely randomized six-system trial previously reported [8], whereas Pen-TMR-TRAD is interpreted as a separate-herd comparator. Each animal contributed one steak to a given assay, with no repeated within-animal measures or random blocks. Accordingly, the one-way ANOVA is equivalent to a mixed model without a random grouping term. When ANOVA indicated significance (*P* < 0.05), Tukey’s multiple comparisons test was applied to identify pairwise differences among treatments. Row-scaled metabolite heatmaps were generated in Python using Matplotlib without row or column clustering. Fatty acid data were expressed as the percentage of total identified fatty acids and n-6:n-3 ratios were calculated as the sum of n-6 divided by the sum of n-3 fatty acids. Individual and grouped fatty acids (SFA, MUFA, and PUFA) were analyzed separately. Metabolite concentrations were reported as µg/100 g wet weight for meat. Individual plant metabolites are reported in Table S2 as µg/100 g dry weight; summed total phenolic concentrations derived from those values are discussed in the text as mg/100 g dry weight. Feed and forage metabolite profiles were summarized descriptively because the samples represented offered materials or shared formulations rather than independent animal-level experimental units. All results are presented as means ± standard error of the mean (SEM), and significance was declared at *P* < 0.05, with trends discussed at 0.05 ≤ *P* ≤ 0.10. Pearson correlation analyses were performed in Python using the SciPy library to assess pairwise associations among meat metabolites at the individual-sample level (*n* = 108). For treatment-mean forage-to-meat biomarker associations (*n* = 7; Fig. 4), Spearman rank correlation analyses were used. Correlation heatmaps and scatter plots, including multi-panel layouts, were generated in Python using the Matplotlib library. Trained descriptive-panel sensory scores were not generated in the present study, and this comparison was not part of the original study design; it became possible because trained-panel data were available for the same finishing systems, and it is presented as an exploratory link between meat chemistry and eating quality. The least-squares treatment means used in Fig. 5 are those reported for the same six finishing systems and the same subsample of carcasses by O’Sullivan et al. [8, 19]. In that work, 14-day aged striploin steaks were cooked to an internal temperature of 71 °C and evaluated at Texas Tech University following American Meat Science Association guidelines by 10 panelists who completed approximately 20 h of training over 2 weeks. Panelists rated 15 flavor and texture attributes on a 100-point scale anchored at 0 (not detectable, extremely dry, or extremely tough) and 100 (extremely intense, extremely juicy, or extremely tender), and least-squares means were estimated with panelist as a random effect. Four attributes (bitter, brown/roasted, fat-like, and metallic) did not differ among systems in that panel and were therefore not carried into the present comparison: an attribute with no treatment-level variation cannot covary with treatment-level chemistry, so including it would contribute noise rather than information. The remaining 11 attributes (bison identity, green-hay-like, liver-like, musty/earthy/humus, oxidized, sour aromatics, bloody/serum, overall sweetness, umami, juiciness, and tenderness) were used. The traditional feedlot treatment (Pen-TMR-TRAD) was not evaluated in that sensory work, so the sensory–chemistry comparison was restricted to the six finishing systems common to both datasets. Seven summary measures of meat chemistry were used, chosen to represent the forage- and grain-associated signatures identified in this study: hippuric acid, catechol, shikimic acid and cinnamoylglycine; long-chain n-3 fatty acids (EPA + DPA + DHA); palmitic acid; and the n-6:n-3 ratio. For each sensory attribute, the published least-squares treatment mean was paired with the treatment mean for each of these measures, and the two sets of treatment means were related by Spearman rank correlation (*n* = 6 treatment means) using the SciPy library in Python; coefficients with nominal (uncorrected) *P* < 0.05 are flagged in Fig. 5. Because these coefficients rest on six treatment means, they are interpreted as exploratory associations rather than as confirmatory tests.

## Results

### Fatty acid composition

The fatty acid compositions of the feeds used across different finishing systems showed notable variation (Table 2). Range-associated feed profiles (Range-DIV, Range-LIM, and the corn-supplemented Range-LIM-CORN composite) contained higher concentrations of alpha-linolenic acid (ALA; C18:3n-3) than pen-based TMR rations, with values of 22.13%, 37.95%, and 13.10%, respectively, compared with values ranging from 4.53% to 5.04% in Pen-TMR-LOW, Pen-TMR-HIGH, and Pen-TMR-TRAD. Pen-FREE-LOW provided free-choice access to both hay and corn and therefore should be interpreted as a mixed-diet pen system rather than as a hay-based comparison. Accordingly, the Pen-FREE-LOW values in Table 2 are best viewed as a dry-matter-weighted treatment-level composite of the offered ingredients rather than as a single consumed ration profile. All pen-based TMR diets exhibited elevated linoleic acid (C18:2n-6) levels, exceeding 50%.

Range-finished bison (Range-DIV, Range-LIM, and Range-LIM-CORN) displayed higher levels of n-3 fatty acids than the pen-finished groups (Table 3; Table S1). Concentrations of alpha-linolenic acid (ALA, C18:3n-3), docosapentaenoic acid (DPA, C22:5n-3), and docosahexaenoic acid (DHA, C22:6n-3) were higher in all range-finished bison (all, *P* < 0.0001), whereas eicosapentaenoic acid (EPA, C20:5n-3) was slightly higher overall in pen-finished animals (*P* < 0.0001; Table 3). Alpha-linolenic acid was roughly four-fold higher in range-finished than in pen-finished bison, and the n-6:n-3 ratios were substantially lower in the range systems (2.8–3.6) than in the pen-fed animals (5.0–7.3; Table 3). The inclusion of corn in the Range-LIM-CORN group reduced total n-3 concentrations and raised the n-6:n-3 ratio relative to the diverse native range (Range-DIV; Table 3).

**Table 3:** Effect of finishing systems on meat fatty acid composition

Fatty acid	Pen-FREE-LOW^1^	Pen-TMR-LOW^1^	Pen-TMR-HIGH^1^	Pen-TMR-TRAD^1^	Range-DIV^1^	Range-LIM^1^	Range-LIM-CORN^1^	*P* value^2,3^
C18:2 (LA)	5.84 ± 0.31^b^	6.25 ± 0.48^b^	7.24 ± 0.49^b^	6.59 ± 0.33^b^	9.19 ± 0.56^a^	9.69 ± 0.43^a^	5.49 ± 0.41^b^	< 0.0001
C18:3 (ALA)	0.56 ± 0.03^de^	0.58 ± 0.03^de^	0.71 ± 0.03CE	0.37 ± 0.04^d^	2.58 ± 0.10^b^	2.87 ± 0.11^a^	0.88 ± 0.06^c^	< 0.0001
C20:4 (AA)	0.42 ± 0.03^bd^	0.44 ± 0.04^bd^	0.51 ± 0.04^bd^	0.25 ± 0.02^ cd^	1.48 ± 0.11^a^	1.54 ± 0.08^a^	0.60 ± 0.05^b^	< 0.0001
C20:5 (EPA)	0.22 ± 0.01^acd^	0.23 ± 0.02^ac^	0.26 ± 0.02^a^	0.26 ± 0.02^a^	0.21 ± 0.01^acd^	0.19 ± 0.01^bcd^	0.17 ± 0.01^bd^	< 0.0001
C22:5 (DPA)	0.74 ± 0.06^bd^	0.77 ± 0.05^bd^	0.91 ± 0.06^b^	0.53 ± 0.03^ cd^	1.97 ± 0.13^a^	1.97 ± 0.09^a^	0.99 ± 0.08^b^	< 0.0001
C22:6 (DHA)	0.18 ± 0.02^cde^	0.20 ± 0.03^bde^	0.24 ± 0.02^be^	0.13 ± 0.01^ cd^	0.45 ± 0.04^a^	0.46 ± 0.03^a^	0.30 ± 0.03^b^	< 0.0001
∑ n-6	8.74 ± 0.51^b^	9.31 ± 0.74^b^	10.80 ± 0.77^b^	9.29 ± 0.47^b^	15.20 ± 1.01^a^	15.62 ± 0.71^a^	8.53 ± 0.65^b^	< 0.0001
∑ n-3	1.69 ± 0.11^bd^	1.79 ± 0.12^bd^	2.13 ± 0.13^b^	1.28 ± 0.04^ cd^	5.22 ± 0.27^a^	5.49 ± 0.21^a^	2.34 ± 0.16^b^	< 0.0001
n-6:n-3 ratio	5.20 ± 0.09^b^	5.15 ± 0.12^b^	5.03 ± 0.11^b^	7.25 ± 0.30^a^	2.88 ± 0.06^d^	2.83 ± 0.05^d^	3.61 ± 0.07^c^	< 0.0001

^1^Treatments: Pen-FREE-LOW = free-choice alfalfa hay, meadow hay, and corn at low stocking density; Pen-TMR-LOW = bison total mixed ration (TMR) at low stocking density; Pen-TMR-HIGH = the same TMR at high stocking density; Pen-TMR-TRAD = traditional TMR at high stocking density (feedlot); Range-DIV = diverse native range; Range-LIM = low-diversity range; Range-LIM-CORN = low-diversity range + corn

^2^*P* values indicate the overall treatment effect across the seven finishing systems; pairwise comparisons were assessed using Tukey’s test when the overall effect was significant. Within a row, treatment means that do not share a common superscript letter differ (*P* < 0.05)

^3^Values are expressed as mean ± standard error of the mean (SEM), as a percentage of total fatty acids; *n* = 16 animals per treatment. Abbreviations: *AA* Arachidonic acid, *ALA* Alpha-linolenic acid, *DHA* Docosahexaenoic acid, *DPA* Docosapentaenoic acid, *EPA* Eicosapentaenoic acid, *LA* Linoleic acid

Linoleic acid (C18:2n-6) concentrations varied considerably across the finishing systems, being highest in Range-DIV and Range-LIM and lowest in Range-LIM-CORN (Table 3). Total n-6 fatty acids followed the same pattern, with the two unsupplemented range systems exceeding all pen-finished groups (Table 3). Pen-based finishing, especially under high-density total mixed ration (TMR) feeding (Pen-TMR-HIGH and Pen-TMR-TRAD), was associated with higher n-6:n-3 ratios (5.0–7.3 vs. 2.8–3.6 in range-finished), driven primarily by lower n-3 fatty acid concentrations rather than by higher absolute n-6 levels. The traditional feedlot system (Pen-TMR-TRAD) exhibited the highest n-6:n-3 ratio and the lowest total n-3 concentration of any treatment, whereas the two bison-TMR systems were intermediate (Table 3). Comparisons involving Pen-TMR-TRAD should nevertheless be interpreted cautiously because that treatment originated from a separate breeding herd.

### Metabolomic profiling

In addition to fatty acid profiling, targeted liquid chromatography–tandem mass spectrometry (LC–MS/MS) analysis of the forages and feed ingredients demonstrated marked variation in phenolic metabolite composition across the offered diets (Table S2). These feed and forage profiles are presented descriptively because they represent sampled materials, shared formulations, or treatment-level composites rather than independent animal-level experimental units. Rangeland forages contained up to 4.2-fold higher summed total phenolic concentrations (329–600 mg/100 g dry weight) than pen-based feeds and supplements (142–262 mg/100 g dry weight). Individual compounds in Table S2 are reported as µg/100 g dry weight, and class totals were calculated as the sum of quantified compounds. Anthocyanins, flavones, and hydroxycinnamic acids were the dominant phenolic classes across the forage and feed samples.

**Fig. 2:**
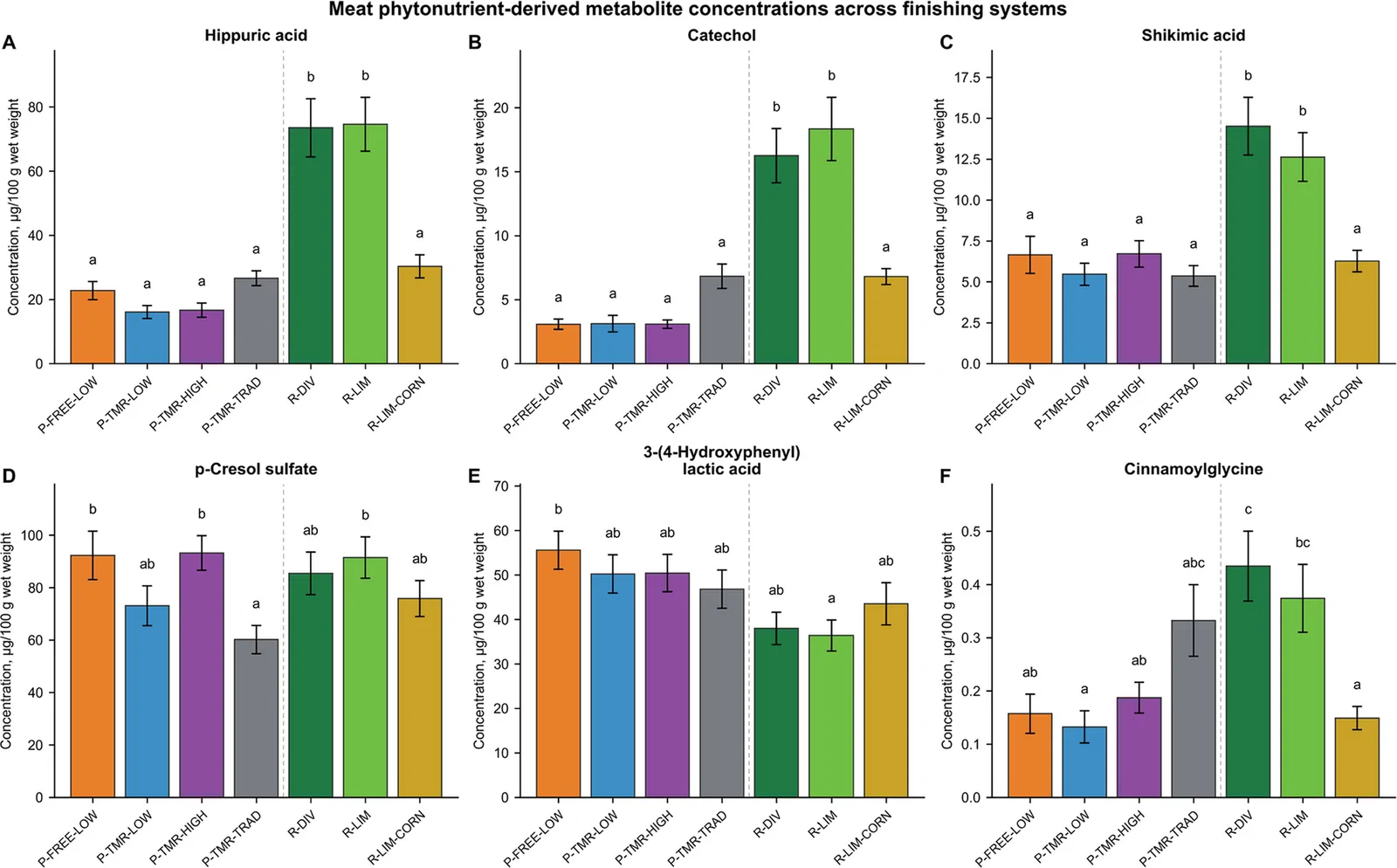
Targeted phytonutrient-derived metabolite concentrations in bison striploins across seven finishing systems. Targeted metabolomic profiling of *M. longissimus lumborum* from bison finished under seven production systems. **A**–**F** Concentrations of hippuric acid (**A**), catechol (**B**), shikimic acid (**C**), p-cresol sulfate (**D**), 3-(4-hydroxyphenyl)lactic acid (**E**), and cinnamoylglycine (**F**). Values are expressed as µg/100 g wet weight and presented as mean ± standard error of the mean (SEM; *n* = 13–18 animals per treatment). Different letters indicate differences among finishing systems (Tukey’s multiple-comparisons test, *P* < 0.05). Treatment abbreviations used in the panels: P-FREE-LOW = Pen-FREE-LOW (free-choice alfalfa hay, meadow hay and corn at low stocking density); P-TMR-LOW = Pen-TMR-LOW (bison total mixed ration [TMR] at low stocking density); P-TMR-HIGH = Pen-TMR-HIGH (the same TMR at high stocking density); P-TMR-TRAD = Pen-TMR-TRAD (traditional TMR at high stocking density, feedlot); R-DIV = Range-DIV (diverse native range); R-LIM = Range-LIM (low-diversity range); R-LIM-CORN = Range-LIM-CORN (low-diversity range plus corn)

**Fig. 3:**
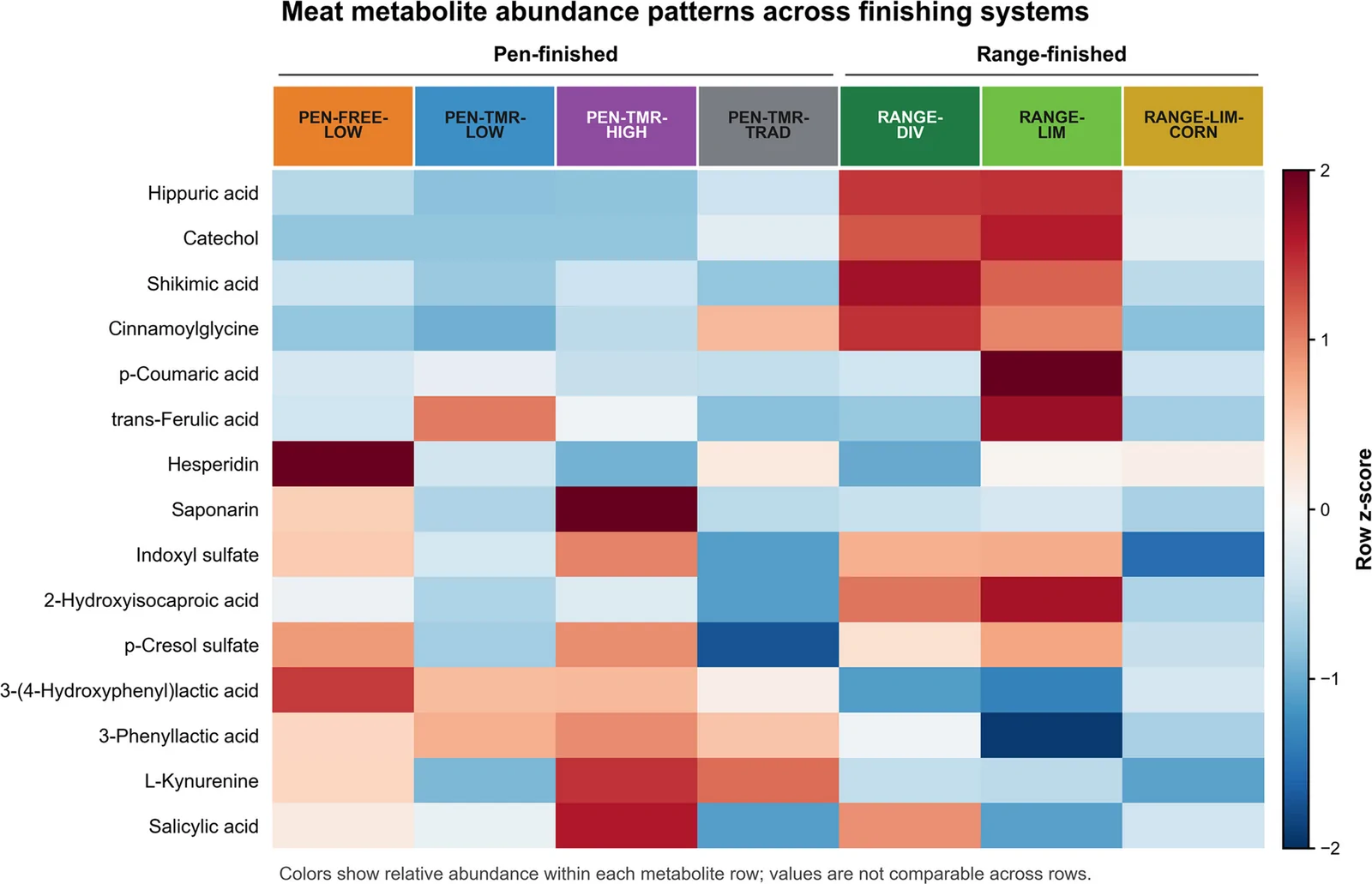
Heatmap of selected targeted metabolite concentrations in bison striploins across seven finishing systems. Row-scaled heatmap showing the relative abundance of targeted metabolites in *M. longissimus lumborum* from bison finished under seven production systems. Metabolite concentrations are displayed as row-scaled z-scores. Columns and rows are shown in a fixed order; no clustering was applied. Treatment means were calculated from detected values and standardized within each metabolite row. Red indicates relatively higher abundance and blue indicates relatively lower abundance. The color pattern summarizes relative treatment-level abundance and is descriptive rather than inferential. Treatment abbreviations: PEN-FREE-LOW, free-choice alfalfa hay, meadow hay and corn at low stocking density; PEN-TMR-LOW and PEN-TMR-HIGH, bison total mixed ration (TMR) at low and high stocking density; PEN-TMR-TRAD, traditional feedlot TMR; RANGE-DIV, diverse native range; RANGE-LIM, low-diversity range; and RANGE-LIM-CORN, low-diversity range plus corn

Targeted metabolomic analysis of bison *M. longissimus lumborum* samples revealed distinct metabolite patterns associated with finishing system and diet composition (Fig. 2). The targeted panel detected 45 metabolites in at least one meat sample. Seven metabolites had reportable overall treatment effects (*P* < 0.05) in Table S2: hippuric acid, catechol, shikimic acid, cinnamoylglycine, p-cresol sulfate, 3-(4-hydroxyphenyl)lactic acid, and saponarin. The most pronounced differences were observed between range-finished and pen-finished systems (Figs. 2 and 3).

Range-finished bison had markedly higher concentrations of phenolic compounds and their microbially derived metabolites (Table S2). Mean hippuric acid concentrations in Range-DIV and Range-LIM were approximately three- to four-fold those of the pen-finished systems, and corn supplementation in range-finished animals (Range-LIM-CORN) reduced hippuric acid to an intermediate level (Table S2).

Catechol, a simple phenol formed by ruminal microbial degradation of dietary plant polyphenols [22, 23], was higher in the range-finished systems (*P* < 0.0001; Table S2). Concentrations in Range-DIV and Range-LIM were several-fold those of all pen-finished systems, and corn supplementation reduced catechol in range-finished animals to a level comparable with the pen-finished groups.

Shikimic acid, an intermediate of the plant shikimate pathway that supplies aromatic amino acids and the secondary metabolites derived from them [24], was markedly elevated in the range-finished systems relative to all pen-finished systems (*P* < 0.0001; Table S2).

Conversely, pen-finished bison exhibited elevated concentrations of 3-(4-hydroxyphenyl)lactic acid, relative to the unsupplemented range-finished systems (*P* = 0.013; Table S2).

Additional metabolites that differed among treatments included p-cresol sulfate, which was lowest in Pen-TMR-TRAD (*P* = 0.013) and did not follow a consistent range-versus-pen pattern (Table S2).

Cinnamoylglycine was elevated in range-finished bison compared with pen-finished animals (*P* < 0.0001; Table S2). Concentrations in Range-DIV and Range-LIM were approximately two-fold those of the pen-finished groups, whereas the traditional feedlot system (Pen-TMR-TRAD) showed intermediate values, and corn supplementation in range-finished animals reduced cinnamoylglycine to a level comparable with the pen-finished systems. The relative abundance of these compounds is summarized in the fixed-order heatmap (Fig. 3).

### Correlation analysis

Correlation analysis of selected treatment-discriminatory meat metabolites showed that several forage-associated biomarkers were positively associated with one another. Hippuric acid was positively correlated with catechol (*r* = 0.91, *P* < 0.001), cinnamoylglycine (*r* = 0.65, *P* < 0.001), and shikimic acid (*r* = 0.74, *P* < 0.001). Catechol was also positively correlated with cinnamoylglycine (*r* = 0.66, *P* < 0.001).

Treatment-level cross-correlation analysis between fatty acid composition and meat metabolite concentrations revealed strong linkages between tissue n-3 status and forage-associated phenolic biomarkers. Total n-3 fatty acid content was positively correlated with hippuric acid (*r* = 0.89, *P* < 0.05), catechol (*r* = 0.82), and cinnamoylglycine (*r* = 0.86, *P* < 0.05), while the n-6:n-3 ratio was inversely correlated with these same metabolites (hippuric acid: *r* = −0.86, *P* < 0.05; catechol: *r* = −0.75). Alpha-linolenic acid (ALA), the primary plant-derived n-3 fatty acid, showed the strongest positive correlations with catechol (*r* = 0.89, *P* < 0.05) and hippuric acid (*r* = 0.86, *P* < 0.05). Conversely, linoleic acid and arachidonic acid, the dominant n-6 fatty acids in grain-based diets, showed negative correlations with these forage-associated biomarkers and positive correlations with 3-(4-hydroxyphenyl)lactic acid.

**Fig. 4:**
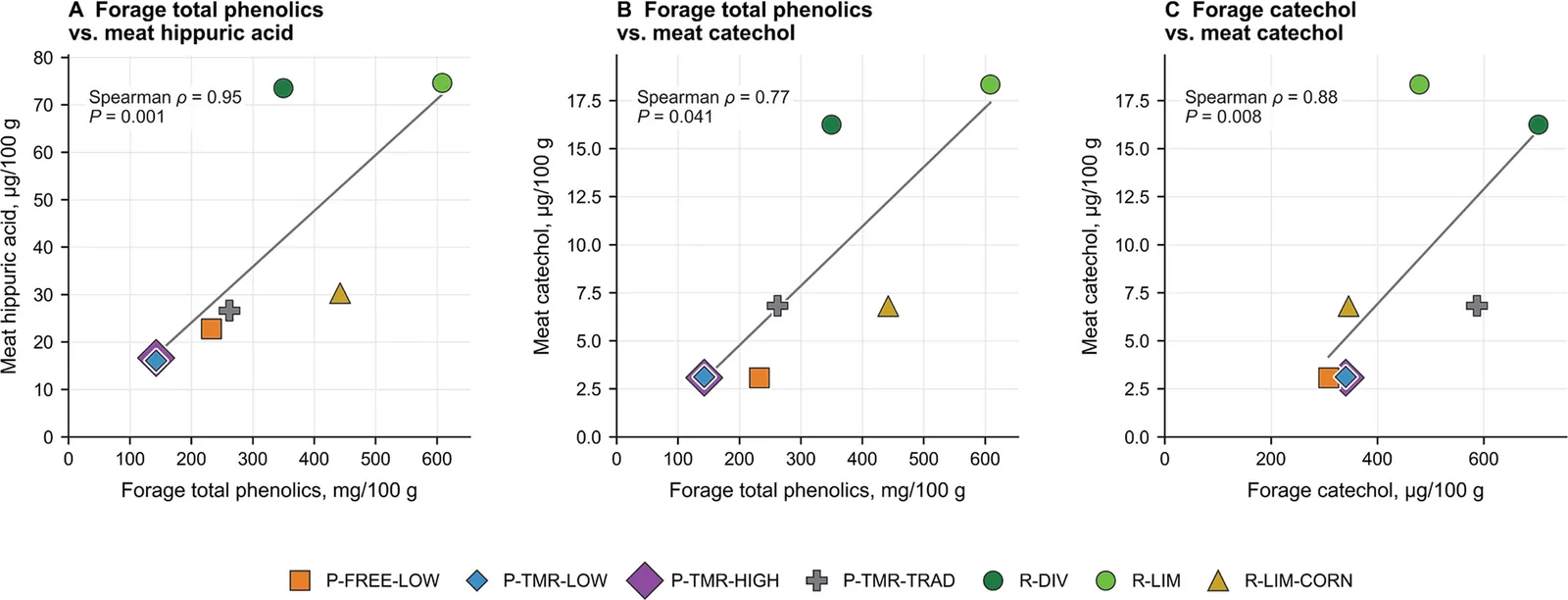
Treatment-level associations between forage phenolic composition and meat phenolic biomarkers across seven finishing systems. **A** forage total phenolics (mg/100 g dry weight) versus meat hippuric acid. **B** Forage total phenolics versus meat catechol. **C** Forage catechol versus meat catechol. Each point is one finishing-system mean (*n* = 7); panel insets report Spearman *ρ* and *P* values. Treatment abbreviations: P-FREE-LOW, free-choice alfalfa hay, meadow hay, and corn at low stocking density; P-TMR-LOW, total mixed ration (TMR) at low stocking density; P-TMR-HIGH, TMR at high stocking density; P-TMR-TRAD, traditional feedlot TMR at high stocking density; R-DIV, diverse native range; R-LIM, low-diversity range; R-LIM-CORN, low-diversity range plus corn. These are biomarker associations, not evidence of direct same-compound transfer

The treatment-level associations in Fig. 4 further underscored the separation between forage- and pen-based systems. Higher forage total phenolics were strongly associated with higher meat hippuric acid (Spearman *ρ* = 0.95, *P* = 0.001; Fig. 4A). Higher forage total phenolics were also associated with higher meat catechol (Spearman *ρ* = 0.77, *P* = 0.041; Fig. 4B), and forage catechol was positively associated with meat catechol (Spearman *ρ* = 0.88, *P* = 0.008; Fig. 4C).

## Discussion

This study found that the finishing system in bison production strongly influences the fatty acid composition and phytonutrient profile of bison meat, with range-finished systems producing meat enriched in n-3 fatty acids and phytonutrients with potential antioxidant and anti-inflammatory properties, compared with bison meat from confinement-based feeding systems. These findings are consistent with previous studies on beef [25], lamb [26], and venison [27], which have found that forage-based diets enhance the omega-3 fatty acid and phytonutrient content in ruminant meat. The results extend the earlier untargeted metabolomics work of van Vliet et al., who reported similar improvements in n-3 fatty acids, phytonutrients, and markers of animal metabolic health in range-finished animals compared to pen-finished animals [13].

The feed and tissue nutrient profiles were broadly aligned across finishing systems, and Fig. 4 highlighted clear associations between forage phenolic composition and meat phenolic biomarkers. The clearest contrast in this study was between range-finished and pen-finished systems, whereas differences attributable to forage diversity within the range-finished systems were comparatively modest given that the limited rangeland still contained an estimated 90 + plant species versus 200 + plant species in the diverse group, arguably making both rangeland treatments quite botanically diverse. Additionally, there were limited differences when the feed in the pen-finished animals was fed as free-choice or as a pre-mixed ration. Because Pen-FREE-LOW provided free-choice access to both hay and corn, the similarity in tissue fatty acid values between Pen-FREE-LOW and Pen-TMR-LOW/Pen-TMR-HIGH is consistent with broadly similar nutrient exposure rather than with differences in feed presentation.

The elevated alpha-linolenic acid (ALA, C18:3n-3) content in range-associated feed profiles (13.10% to 37.95%) compared to pen-based TMR rations (4.53% to 5.04%) likely contributed to higher tissue concentrations of DPA and DHA in the range-finished animals [28, 29]. Notably, EPA (C20:5n-3) concentrations were slightly higher overall in pen-finished bison, with significant differences in some pen-versus-range comparisons, in contrast to the pattern observed for the other long-chain n-3 PUFAs (Table 3; Table S1). This pattern may reflect differences in the efficiency of the desaturation-elongation pathway between the feeding systems. In range-finished animals with abundant dietary ALA, the conversion pathway proceeds efficiently through EPA to the longer-chain products DPA and DHA [30], resulting in lower EPA accumulation, as it serves primarily as a metabolic intermediate. Conversely, in grain-fed animals with limited ALA substrate, the reduced flux through this pathway may result in EPA accumulation rather than continued elongation. Additionally, the high linoleic acid content of grain-based diets competes with ALA for Δ6-desaturase, the rate-limiting enzyme in long-chain PUFA synthesis [31], which may further alter the balance of the pathway intermediates. This interpretation is consistent with the substantially higher DPA and DHA concentrations in range-finished bison, indicating that the elongation pathway is likely more complete in range-finished animals than in grain-supplemented animals. These findings highlight the complexity of fatty acid deposition in tissues and suggest that individual fatty acid concentrations should be interpreted within the context of the entire biosynthetic pathway. Range-finished bison exhibited n-6:n-3 ratios between 2.8 and 3.6, which are substantially lower than those of pen-finished animals and approach the ≤ 4:1 ratio that has been proposed as a desirable dietary target [32]. That ratio is a proposed target rather than a formal dietary recommendation, and its usefulness continues to be debated. Feedlot-finished animals fed a traditional TMR exhibited a ratio of 7.25, which was higher than that of other pen-finished animals (5.0 to 5.2). The intermediate n-6:n-3 ratios observed in pen-finished animals (excluding traditional TMR) can be attributed to their diet composition, which contained proportionally more hay and less grain than traditional TMR-fed animals, resulting in a more favorable fatty acid profile that falls between that of range-finished and traditional TMR-fed bison. Western diets typically contain n-6:n-3 ratios ranging from 15:1 to 20:1 [33], which are associated with increased inflammatory responses and cardiometabolic risk [34, 35].

Our findings broadly align with the literature for beef, particularly regarding studies focusing on the enrichment of n-3 fatty acids through dietary modifications in cattle finding higher levels in forage-finished beef [36, 37]. Additionally, a recent profiling study of > 300 samples of grass-fed and grain-fed beef from commercial North American production systems found that grass-fed beef, particularly those finished on biodiverse pastures, was enriched in n-3 fatty acids compared to grain-fed beef [38]. Importantly, human randomized controlled trials (RCTs) examining fatty acid profiles of ruminant meat have found that consumption of grass-fed meat enriched with n-3 fatty acids can increase circulating n-3 fatty acid concentrations in consumers [7, 39]. These RCTs found that the intake of ruminant meat with enhanced n-3 content leads to measurable increases in plasma and erythrocyte n-3 fatty acid levels in humans [7], reflecting improved bioavailability and incorporation of these beneficial lipids into circulation. This evidence supports the role of ruminant meat as a dietary source capable of modulating circulating fatty acid profiles, which may have downstream effects on health-related biomarkers [40]. Thus, the lower n-6:n-3 ratios in range-finished and pen-finished bison meat with proportionally more forage and less corn than a traditional TMR, suggests that the consumption of such products could contribute to an improved dietary fatty acid balance in human populations; however, our data do not indicate that pen-finished bison meat is inherently unhealthy to consume, particularly given that human intervention studies have not found the same short-term adverse effects for ruminant-derived trans fatty acids as for industrial trans fats in healthy adults [39]. Notably, the n-6:n-3 ratio in the pen-finished systems other than the traditional feedlot TMR remained lower (5.0–5.2) than the ratio measured in the traditional feedlot group (7.25), indicating that pen finishing with proportionally more hay and less grain does not reproduce the fatty acid profile of a conventional feedlot ration.

More broadly, diets rich in n-3 fatty acids are consistently associated with improved metabolic health [41, 42]. Omega-3-rich dietary patterns have been linked to reduced systemic inflammation, improved lipid profiles [43], enhanced insulin sensitivity [44], and favorable modulation of cardiovascular risk factors [45]. These effects are thought to arise from their capacity to influence cell membrane fluidity [46], metabolism-related gene expression [47], and anti-inflammatory signaling pathways [48]. For example, higher dietary n-3 intake and lower n-6:n-3 ratios have been linked to reduced systemic inflammation [49], as evidenced by lower concentrations of inflammatory markers such as C-reactive protein, interleukin-6, and tumor necrosis factor-alpha [50].

Phytonutrient profiling revealed that the finishing system also influences the broader metabolite signature of bison meat. The metabolite patterns observed in this study reflect the fundamental differences in dietary inputs and their biotransformation through ruminal and host metabolism [51]. Range-finished animals consuming a diverse array of forages ingest a wide array of plant secondary metabolites, including flavonoids, phenolic acids, and other polyphenolic compounds. Targeted phenolic profiling of the forages used in this study confirmed this pattern: rangeland forages contained summed total phenolic concentrations of 329–600 mg/100 g dry weight compared with 142–262 mg/100 g dry weight in pen-based feeds (Table S2). These totals were calculated from the individual compounds reported in Table S2 as µg/100 g dry weight. Notably, the feed corn sample contained the lowest summed total phenolics of any feed or forage analyzed, consistent with the attenuated phenolic biomarker profile observed in corn-supplemented systems [14].

Forage phytonutrients may be deposited more directly in tissue or transformed by the ruminal microbiota, yielding metabolites such as hippuric acid (from benzoic acid and other aromatic precursors) and catechol (from plant phenolics) [22, 23], whereas shikimic acid, an intermediate of the plant shikimate pathway [24], more directly reflects plant aromatic biosynthesis pathways [52]. Thus, the enrichment of hippuric acid, catechol, and cinnamoylglycine in range-finished animals reflects the consumption and microbial transformation of plant polyphenols, which are abundant in diverse pasture systems but are lower in grain-based concentrates [53]. In particular, hippuric acid is formed through the conjugation of benzoic acid, derived from dietary polyphenols, with glycine in the liver [54]. In non-uremic humans, higher levels of hippuric acid are associated with improved gut microbial diversity and a lower risk of metabolic syndromes [55, 56]. In ruminants, hippuric acid is considered a potential biomarker of grass-finishing [57] and has also been discussed in relation to animal metabolic health [14].

Cinnamoylglycine was elevated approximately two-fold in the range-finished bison compared to the pen-finished bison. Cinnamoylglycine is a microbially derived metabolite formed from cinnamic acid-related aromatics and subsequently conjugated with glycine in the liver [58]. Higher forage intake increases ruminant exposure to phenylpropanoids and other aromatic substrates, resulting in elevated levels of cinnamoylglycine, which can potentially also serve as a biomarker of forage-driven aromatic metabolism [59]. These findings are in line with previous work on bison, which found that cinnamoylglycine was ~ threefold higher in range-finished than in pen-finished bison on feed [13]. In ruminants, this pattern is generally interpreted as indicating greater phenolic exposure and has been linked, in broader pasture/plant-rich dietary contexts, to metabolic signatures consistent with improved animal metabolic health (e.g., oxidative stress and mitochondrial function pathways) [15]. In humans, cinnamoylglycine is correlated with diet quality and is inversely associated with the risk of type 2 diabetes [60]. Whether dietary exposure to cinnamoylglycine through animal food directly impacts circulating levels in humans is currently unknown. Importantly, forage-derived phytochemicals may help limit lipid oxidation in meat, but whether this meaningfully affects downstream circulating n-3 fatty acid levels in consumers remains unclear [61, 62].

In contrast, pen-finished systems relying on grain-based TMR or free-choice grain supplementation produce different metabolic signatures. Elevated concentrations of 3-(4-hydroxyphenyl)lactic acid in pen-finished bison represent a metabolite signature associated with grain-based feeding and altered ruminal fermentation patterns [13]. This metabolite is formed when bacteria transaminate tyrosine to 4-hydroxyphenylpyruvate and then reduce it to 4-hydroxyphenyllactate through an aromatic lactate dehydrogenase, an activity characterized in gut commensals [63] and in lactic acid bacteria, whose output of 4-hydroxyphenyllactate varies with the supply of fermentable carbohydrate [64]. High-concentrate diets shift the ruminal community toward the lactate-producing populations that carry this activity [59], whereas forage-based diets favor a different community structure. This comparison also shows how energy supplementation on a low-diversity pasture compares with a high-diversity pasture without supplementation [65, 66]. The inclusion of corn supplementation on rangelands resulted in intermediate fatty acid profiles, with n-3 concentrations lower than those in exclusively range-finished bison but higher than those in the pen-finished groups. Additionally, hippuric acid showed a more intermediate profile, with levels lower than those in bison finished exclusively on forages but higher than those in bison finished on grain-based concentrates. This finding indicates that, even within forage-based systems, dietary composition exerts measurable effects on meat nutritional composition (Table S2). Producers seeking to optimize meat nutritional composition while maintaining some degree of supplementation should consider the trade-offs between animal performance and nutritional attributes when formulating finishing diets. Additionally, producers can consider feeding grains on pastures during limited times of the year (e.g., during the winter) to ensure adequate energy intake as opposed to using it as a finishing ration. Furthermore, while high stocking densities can increase animal stress, which may alter metabolism [67], our results indicated no significant differences in meat fatty acid or metabolite profiles between the high and low stocking density pen systems.

The treatment-level patterns across range- and pen-based systems in the fixed-order heatmap (Fig. 3) are consistent with reports that metabolomic profiles can help distinguish products from different production systems in beef and goat milk [68, 69]. The observed contrasts between range- and pen-finished systems and the effect of corn supplementation further show that management practices are reflected in the measured metabolite profile. These findings add to evidence that finishing practices alter meat metabolite composition, with potential implications for meat quality and animal or human health outcomes [70], although the latter require further study.

The correlation analyses further highlight the integrated nature of the nutritional differences between finishing systems. The strong intercorrelations among hippuric acid, catechol, and cinnamoylglycine suggest that these metabolites rise together across finishing systems and are likely influenced by both ruminal and host metabolism. Together with the treatment-level forage-to-meat associations (Fig. 4), these patterns reinforce the separation between forage- and grain-associated metabolic signatures. Importantly, the cross-correlation between fatty acid profiles and metabolite concentrations indicates that these two dimensions of meat composition are not independent but rather co-vary as a function of dietary intake. Range-finished animals with favorable n-3 fatty acid profiles also carried higher concentrations of phytonutrient-related metabolites (Figs. 2 and 4), whereas pen-finished animals with elevated n-6:n-3 ratios showed lower levels across many phytonutrient metabolites. From a consumer perspective, these findings suggest that range-finished bison meat differs from pen-finished meat not only in n-3 enrichment but also in a broader set of metabolites associated with forage intake and polyphenolic metabolism. These integrated profiles are consistent with broader literature linking higher n-3 and phytochemical intakes with reduced inflammation and improved cardiometabolic health outcomes [71], although those effects were not directly tested here.

### Integrating sensory and chemical signatures

**Fig. 5:**
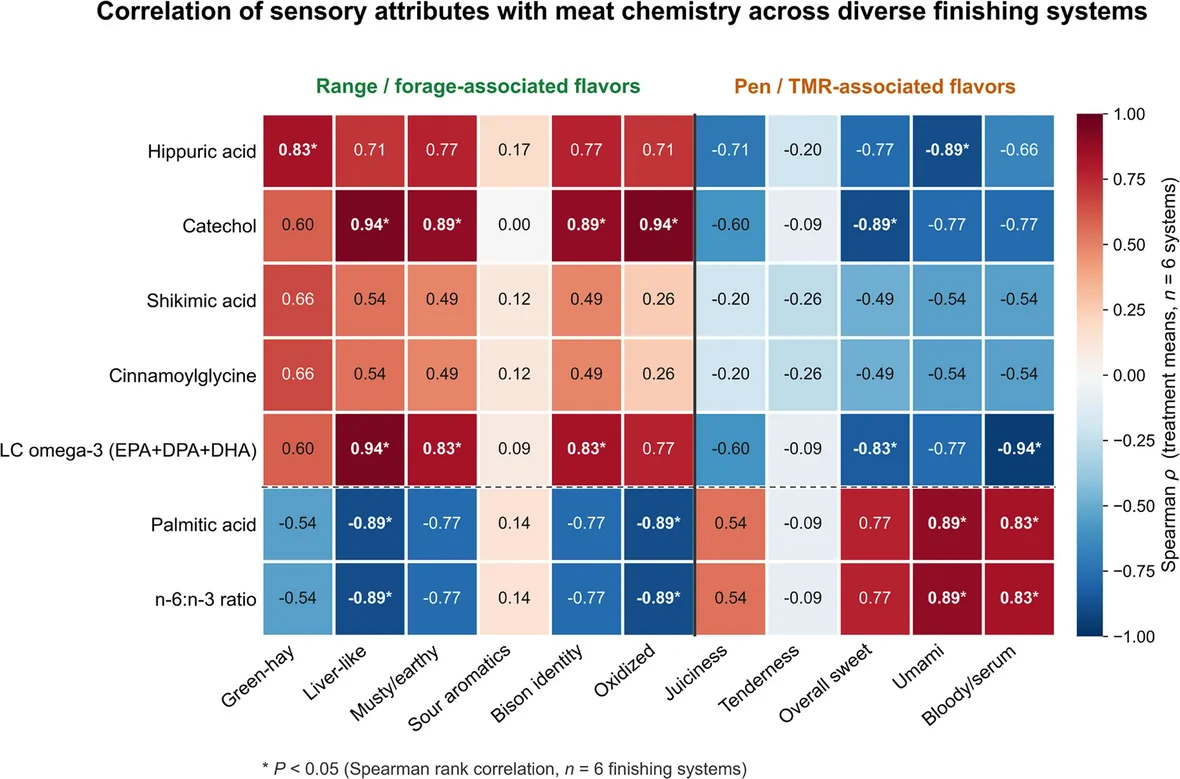
Treatment-level Spearman correlations (*ρ*) between trained descriptive-panel sensory attributes and meat chemistry across the six diverse bison finishing systems common to both datasets (*n* = 6 system means). Treatment abbreviations: P-FREE-LOW = Pen-FREE-LOW (free-choice alfalfa hay, meadow hay, and corn at low stocking density); P-TMR-LOW = Pen-TMR-LOW (bison total mixed ration [TMR] at low stocking density); P-TMR-HIGH = Pen-TMR-HIGH (the same TMR at high stocking density); R-DIV = Range-DIV (diverse native range); R-LIM = Range-LIM (low-diversity range); R-LIM-CORN = Range-LIM-CORN (low-diversity range plus corn). Additional abbreviations used in the heatmap: *LC* Long-chain, *EPA* Eicosapentaenoic acid, *DPA* Docosapentaenoic acid, *DHA* Docosahexaenoic acid. Sensory least-squares means are from a trained panel evaluating 14-day aged striploin steaks; meat phenolic metabolites and fatty acids are from the present study. Rows progress from range-/forage-associated chemistry (plant phenolics, long-chain omega-3) to pen-/TMR-associated chemistry (palmitic acid, n-6:n-3 ratio); columns group forage-associated flavors (left) and pen/TMR-associated flavors (right). Red denotes positive and blue negative correlation; ^*^P < 0.05. Forage-associated flavors track phenolic/omega-3 enrichment, whereas juicy, fatty, and umami attributes track the fat-associated chemistry of grain/TMR finishing

To connect these production-driven chemical differences to eating quality, we related the meat metabolite and fatty-acid gradients measured here to trained descriptive-panel sensory scores reported for the same six diverse finishing systems [8] (Fig. 5). Sensory attributes that were most intense in range- or forage-finished steaks (green-hay, liver-like, musty or earthy, oxidized, and overall bison-identity character) were positively correlated across systems with the plant-derived phenolics (hippuric acid, catechol, shikimic acid, cinnamoylglycine) and long-chain omega-3 fatty acids that were likewise elevated in range-finished bison (Spearman *ρ* up to 0.94), whereas sour-aromatic character was unrelated to these compounds (*ρ* ≤ 0.17). Conversely, attributes that were most intense in pen- or TMR-finished steaks (juiciness, overall sweetness, umami, and bloody or serum character) were positively correlated with palmitic acid and the n-6:n-3 ratio and negatively correlated with the phenolic biomarkers, whereas tenderness was not associated with either chemical group (|*ρ*| ≤ 0.26). This helps explain the perception that forage-finished bison carries a grassy or pasture character, while grain- or TMR-finished bison is perceived as juicier, fattier, and more umami [72–75]. For producers, this points to a link between the finishing decision and the flavor profile of the resulting product; because the panel was a trained descriptive panel rather than a consumer preference panel, these data describe how the meat is perceived and not which product consumers would choose. Because these correlations are based on six treatment-mean values, they are interpreted as illustrative associations rather than causal proof and warrant confirmation in integrated sensory–metabolomic studies.

From a sustainability perspective, the findings suggest that well-managed range-finished systems can produce meat with enhanced nutritional profiles while potentially supporting ecosystem function through the maintenance of native grasslands and promotion of biodiversity, as well as associated ecosystem services [76]. The diverse plant communities in range-finished systems may enhance the phytochemical content of meat while also contributing to soil health, carbon sequestration, and habitat provision [14, 77].

This study has several limitations. First, the animals in the conventional TMR feedlot treatment originated from a separate breeding herd, so differences involving Pen-TMR-TRAD cannot be attributed exclusively to finishing system. Second, while the limited diversity rangeland treatment was more limited in plant diversity compared to the diverse rangeland, the “limited” diversity rangeland still contained approximately 90 plant species. Although it was dominated by a few meadow grasses, it was already highly diverse and in far excess of the diversity found in many pastures. This high baseline diversity explains why the metabolomics and phytonutrient profiles for the “diverse” forage group did not consistently exceed, and in some respects were even less than, those of the “limited” group. As a result, the “limited” range treatment here should not be interpreted as a typical low-diversity pasture dominated by a few species of plants. Third, this study did not directly evaluate consumer sensory preferences or willingness to pay for meat from different finishing systems, both of which are important factors for market viability [78]. However, carcass characteristics, proximate composition, and palatability outcomes from these same finishing systems were reported previously by O’Sullivan et al. [8]. Fourth, feed and forage composition was characterized from offered materials and shared ration formulations rather than from replicated, animal-level samples, and individual feed intake was not measured; the dietary contrasts therefore describe the systems as offered rather than what each animal actually consumed. Fifth, the forage-to-meat and sensory-to-chemistry relationships in Figs. 4 and 5 rest on seven and six treatment means, respectively, and are exploratory associations that cannot establish causality or support inference at the level of individual animals. Sixth, metabolite profiling used a targeted panel, so compounds outside that panel were not measured and the summed phenolic values reported here are limited to the quantified compounds. Seventh, all animals originated from a single commercial source herd finished in one region during a single production cycle, so the magnitude of the differences reported here may not generalize to other genetics, plant communities, or years. Finally, while this study investigated phytonutrient and fatty acid profiles, it is possible that broader nutritional differences exist between treatment groups in terms of minerals, vitamins, and protein content. Elaidic acid was quantified in both feed samples (Table 2) and meat (Table S1), whereas vaccenic acid and rumenic acid were quantified in meat and are also reported in Table S1; the focus in this work was on the n-3 and n-6 fatty acid families and phytonutrients as they tend to provide the greatest contrast between forage and grain-based finishing systems [5].

## Conclusions

This study found that finishing environment is a major factor shaping the fatty acid and phytonutrient composition of bison meat, although comparisons involving the conventional TMR feedlot treatment should be interpreted cautiously because that group originated from a separate breeding herd. Range-finished systems enhanced n-3 polyunsaturated fatty acids and polyphenol-derived metabolites, whereas the conventional TMR feedlot treatment produced the highest n-6:n-3 ratios and the lowest phytochemical content, while corn supplementation on pasture yielded an intermediate profile. These findings do not indicate that pen-finished bison meat is unhealthy; they show that range-finished bison meat contained more n-3 fatty acids, had a lower n-6:n-3 ratio, and carried higher concentrations of forage-derived phenolic metabolites. Whether these compositional differences translate into measurable health outcomes in consumers was not tested here. Relating these chemical gradients to trained descriptive sensory-panel scores from the same finishing systems further suggested, at an exploratory level based on six treatment means, that the forage-associated phenolic and n-3 fatty-acid signatures track the green, earthy character of range-finished bison, whereas pen-finishing tracks juiciness, sweetness, and umami attributes. Future research should translate these compositional differences into measurable health outcomes through human feeding trials, confirm these chemical–sensory associations in integrated sensory–metabolomic studies, and evaluate the economic feasibility of range-finishing systems.

## Supplementary Information

Additional file 1: Table S1. Fatty acid composition of bison *M. longissimus lumborum* across seven finishing systems. Table S2. Targeted metabolite concentrations in bison *M. longissimus lumborum* (A) and in the feeds and forages offered in each finishing system (B), determined by LC–MS/MS.

## Data Availability

All data generated or analyzed during this study are included in this published article and its supplementary information files. Fatty acid composition data are provided in Table S1, and individual plant and meat metabolite concentrations are provided in Table S2. Additional datasets used and analyzed during the current study are available from the corresponding author on reasonable request.

## Abbreviations

AA: Arachidonic acid
ADF: Acid detergent fiber
ALA: Alpha-linolenic acid
ANOVA: Analysis of variance
ARS: Agricultural Research Service
BHT: Butylated hydroxytoluene
CAD: Collision gas
CE: Collision energy
CLA: Conjugated linoleic acid
CP: Crude protein
CXP: Collision cell exit potential
DHA: Docosahexaenoic acid
DM: Dry matter
DPA: Docosapentaenoic acid
EPA: Eicosapentaenoic acid
FAME: Fatty acid methyl ester
GC-FID: Gas chromatography–flame ionization detection
HCW: Hot carcass weight
HSS: High Strength Silica
LA: Linoleic acid
LC–MS/MS: Liquid chromatography–tandem mass spectrometry
MRM: Multiple reaction monitoring
MTBE: Methyl tert-butyl ether
MUFA: Monounsaturated fatty acid
NDF: Neutral detergent fiber
NEm: Net energy for maintenance
NIFA: National Institute of Food and Agriculture
PUFA: Polyunsaturated fatty acid
Q0D: Q0 dissociation
QC: Quality control
RCT: Randomized controlled trial
RFV: Relative feed value
S/N: Signal-to-noise
SARE: Sustainable Agriculture Research and Education
SEM: Standard error of the mean
SFA: Saturated fatty acid
SPE: Solid-phase extraction
TDN: Total digestible nutrients
TMR: Total mixed ration
UPLC: Ultra-performance liquid chromatography
USDA: United States Department of Agriculture

## References

[1] Juárez M, Lam S, Bohrer BM, Dugan MER, Vahmani P, Aalhus J, et al. Enhancing the nutritional value of red meat through genetic and feeding strategies. Foods. 2021;10:872. 10.3390/foods10040872. PMC8073878 33923499

[2] Lourenço M, Ramos-Morales E, Wallace RJ. The role of microbes in rumen lipolysis and biohydrogenation and their manipulation. Animal. 2010;4:1008–23. 10.1017/S175173111000042X. 22444606

[3] Acamovic T, Brooker JD. Biochemistry of plant secondary metabolites and their effects in animals. Proc Nutr Soc. 2005;64:403–12. 10.1079/PNS2005449. 16048675

[4] Ponnampalam EN, Kearns M, Kiani A, Santhiravel S, Vahmani P, Prache S, et al. Enrichment of ruminant meats with health enhancing fatty acids and antioxidants: feed-based effects on nutritional value and human health aspects – invited review. Front Anim Sci. 2024;5:1329346. 10.3389/fanim.2024.1329346.

[5] Krusinski L, Maciel ICF, van Vliet S, Ahsin M, Adams J, Lu G, et al. Fatty acids and secondary metabolites can predict grass-finished beef and supplemental cattle feeds. npj Sci Food. 2024;8:73. 10.1038/s41538-024-00315-5. PMC11452727 39367030

[6] Bishehkolaei M, Pathak Y. Influence of omega n-6/n-3 ratio on cardiovascular disease and nutritional interventions. Hum Nutr Metab. 2024;37:200275. 10.1016/j.hnm.2024.200275.

[7] McAfee AJ, McSorley EM, Cuskelly GJ, Fearon AM, Moss BW, Beattie JAM, et al. Red meat from animals offered a grass diet increases plasma and platelet n-3 PUFA in healthy consumers. Br J Nutr. 2011;105:80–9. 10.1017/S0007114510003090. 20807460

[8] O’Sullivan LM, Anema RF, Weldy GT, Carpenter BJ, Hernandez MS, Underwood KR, et al. Influence of diverse finishing systems on carcass characteristics, proximate composition, and meat quality attributes of striploins from bison heifers. Front Ecol Evol. 2025;13:1686159. 10.3389/fevo.2025.1686159.

[9] Tielkes S, Altmann BA. The sustainability of bison production in North America: a scoping review. Sustainability. 2021;13:13527. 10.3390/su132413527.

[10] Weldy GT, O’Sullivan LM, Blair AD. Recent advances in meat quality of North American bison – a review. Meat Sci. 2026. 10.1016/j.meatsci.2026.110090. 41875695

[11] Wang B, Zhao X, Li Z, Luo H, Zhang H, Guo Y, et al. Changes of metabolites and gene expression under different feeding systems associated with lipid metabolism in lamb meat. Foods. 2021;10:2612. 10.3390/foods10112612. PMC8619812 34828897

[12] Carrillo JA, He Y, Li Y, Liu J, Erdman RA, Sonstegard TS, et al. Integrated metabolomic and transcriptome analyses reveal finishing forage affects metabolic pathways related to beef quality and animal welfare. Sci Rep. 2016;6:25948. 10.1038/srep25948. PMC4869019 27185157

[13] van Vliet S, Blair AD, Hite LM, Cloward J, Ward RE, Kruse C, et al. Pasture-finishing of bison improves animal metabolic health and potential health-promoting compounds in meat. J Anim Sci Biotechnol. 2023;14:49. 10.1186/s40104-023-00843-2. PMC10067211 37004100

[14] Ahsin M, Poore MH, Rogers J, Franzluebbers A, Young SN, Kronberg SL, et al. Soil and pasture health underlie improved beef nutrient density determined by untargeted metabolomics in Southern US grass finished beef systems. npj Sci Food. 2025;9:151. 10.1038/s41538-025-00471-2. PMC12290049 40707531

[15] Ahsin M, Matarneh SK, Thornton KJ, Kronberg S, Amir M, van Vliet S. Phenolic compounds and derivatives in ruminant meat and milk: a systematic review. J Agric Food Chem. 2025;73:29961–82. 10.1021/acs.jafc.5c06118. PMC12679856 41235804

[16] Nunes CdosR, Arantes MB, Pereira SMdeF, Cruz LLda, Passos MdeS, Moraes LPde, et al. Plants as sources of anti-inflammatory agents. Molecules. 2020;25:3726. 10.3390/molecules25163726. PMC7465135 32824133

[17] Wink M. Medicinal plants: a source of anti-parasitic secondary metabolites. Molecules. 2012;17:12771–91. 10.3390/molecules171112771. PMC6268567 23114614

[18] Swallah MS, Sun H, Affoh R, Fu H, Yu H. Antioxidant potential overviews of secondary metabolites (polyphenols) in fruits. Int J Food Sci. 2020;2020:9081686. 10.1155/2020/9081686. PMC7229537 32455130

[19] O’Sullivan LMH. Role of production systems on carcass and palatability attributes of bison [PhD thesis]. Brookings (SD): South Dakota State University; 2024. https://openprairie.sdstate.edu/etd2/1133.

[20] O’Fallon JV, Busboom JR, Nelson ML, Gaskins CT. A direct method for fatty acid methyl ester synthesis: application to wet meat tissues, oils, and feedstuffs. J Anim Sci. 2007;85:1511–21. 10.2527/jas.2006-491. 17296772

[21] Broadhurst D, Goodacre R, Reinke SN, Kuligowski J, Wilson ID, Lewis MR, et al. Guidelines and considerations for the use of system suitability and quality control samples in mass spectrometry assays applied in untargeted clinical metabolomic studies. Metabolomics. 2018;14:72. 10.1007/s11306-018-1367-3. PMC5960010 29805336

[22] González-Barrio R, Truchado P, García-Villalba R, Hervás G, Frutos P, Espín JC, et al. Metabolism of oak leaf ellagitannins and urolithin production in beef cattle. J Agric Food Chem. 2012;60:3068–77. 10.1021/jf300718k. 22375726

[23] Martin AK. The origin of urinary aromatic compounds excreted by ruminants. 3. The metabolism of phenolic compounds to simple phenols. Br J Nutr. 1982;48:497–507. 10.1079/bjn19820135. 7171537

[24] Maeda H, Dudareva N. The shikimate pathway and aromatic amino acid biosynthesis in plants. Annu Rev Plant Biol. 2012;63:73–105. 10.1146/annurev-arplant-042811-105439. 22554242

[25] Evans N, Cloward J, Ward RE, van Wietmarschen HA, van Eekeren N, Kronberg SL, et al. Pasture-finishing of cattle in Western U.S. rangelands improves markers of animal metabolic health and nutritional compounds in beef. Sci Rep. 2024;14:20240. 10.1038/s41598-024-71073-3. PMC11364752 39215122

[26] Ponnampalam EN, Butler KL, Jacob RH, Pethick DW, Ball AJ, Hocking Edwards JE, et al. Health beneficial long chain omega-3 fatty acid levels in Australian lamb managed under extensive finishing systems. Meat Sci. 2014;96:1104–10. 10.1016/j.meatsci.2013.04.007. 23643471

[27] Kilar J, Kasprzyk A. Fatty acids and nutraceutical properties of lipids in fallow deer (*Dama dama*) meat produced in organic and conventional farming systems. Foods. 2021;10:2290. 10.3390/foods10102290. PMC8534888 34681339

[28] Scollan ND, Dannenberger D, Nuernberg K, Richardson I, MacKintosh S, Hocquette JF, et al. Enhancing the nutritional and health value of beef lipids and their relationship with meat quality. Meat Sci. 2014;97:384–94. 10.1016/j.meatsci.2014.02.015. 24697921

[29] Ponnampalam EN, Sinclair AJ, Holman BWB. The sources, synthesis and biological actions of omega-3 and omega-6 fatty acids in red meat: an overview. Foods. 2021;10:1358. 10.3390/foods10061358. PMC8230916 34208278

[30] Noci F, Monahan FJ, French P, Moloney AP. The fatty acid composition of muscle fat and subcutaneous adipose tissue of pasture-fed beef heifers: influence of the duration of grazing. J Anim Sci. 2005;83:1167–78. 10.2527/2005.8351167x. 15827261

[31] Zhang JY, Kothapalli KSD, Brenna JT. Desaturase and elongase limiting endogenous long chain polyunsaturated fatty acid biosynthesis. Curr Opin Clin Nutr Metab Care. 2016;19:103–10. 10.1097/MCO.0000000000000254. PMC4768719 26828581

[32] Simopoulos AP. The importance of the ratio of omega-6/omega-3 essential fatty acids. Biomed Pharmacother. 2002;56:365–79. 10.1016/s0753-3322(02)00253-6. 12442909

[33] Molendi-Coste O, Legry V, Leclercq IA. Why and how meet n-3 PUFA dietary recommendations? Gastroenterol Res Pract. 2011. 10.1155/2011/364040. PMC3004387 21197079

[34] Gonzalez-Becerra K, Barron-Cabrera E, Muñoz-Valle JF, Torres-Castillo N, Rivera-Valdes JJ, Rodriguez-Echevarria R, et al. A balanced dietary ratio of n-6:n-3 polyunsaturated fatty acids exerts an effect on total fatty acid profile in RBCs and inflammatory markers in subjects with obesity. Healthcare. 2023;11:2333. 10.3390/healthcare11162333. PMC10454033 37628530

[35] von Schacky C, Harris WS. Cardiovascular benefits of omega-3 fatty acids. Cardiovasc Res. 2007;73:310–5. 10.1016/j.cardiores.2006.08.019. 16979604

[36] Urrutia O, Mendizabal JA, Alfonso L, Soret B, Insausti K, Arana A. Adipose tissue modification through feeding strategies and their implication on adipogenesis and adipose tissue metabolism in ruminants. Int J Mol Sci. 2020;21:3183. 10.3390/ijms21093183. PMC7246642 32365995

[37] Duckett SK, Neel JPS, Lewis RM, Fontenot JP, Clapham WM. Effects of forage species or concentrate finishing on animal performance, carcass and meat quality. J Anim Sci. 2013;91:1454–67. 10.2527/jas.2012-5914. 23345568

[38] Varre JV, Statham T, Smith AF, Ahsin M, Cloward J, Herrera MC, et al. Nutritional composition of beef: a comparison of commercial North American grass- and grain-finishing systems. J Anim Sci. 2026;104:skaf436. 10.1093/jas/skaf436. PMC13012854 41414663

[39] Radtke T, Schmid A, Trepp A, Dähler F, Coslovsky M, Eser P, et al. Short-term effects of trans fatty acids from ruminant and industrial sources on surrogate markers of cardiovascular risk in healthy men and women: a randomized, controlled, double-blind trial. Eur J Prev Cardiol. 2017;24:534–43. 10.1177/2047487316680691. 27899529

[40] Bessa RJB, Alves SP, Santos-Silva J. Constraints and potentials for the nutritional modulation of the fatty acid composition of ruminant meat. Eur J Lipid Sci Technol. 2015;117:1325–44. 10.1002/ejlt.201400468.

[41] Robinson LE, Buchholz AC, Mazurak VC. Inflammation, obesity, and fatty acid metabolism: influence of n-3 polyunsaturated fatty acids on factors contributing to metabolic syndrome. Appl Physiol Nutr Metab. 2007;32:1008–24. 10.1139/H07-087. 18059573

[42] Zivkovic AM, Telis N, German JB, Hammock BD. Dietary omega-3 fatty acids aid in the modulation of inflammation and metabolic health. Calif Agric. 2011;65:106–11. 10.3733/ca.v065n03p106. PMC4030645 24860193

[43] Yang LG, Song ZX, Yin H, Wang YY, Shu GF, Lu HX, et al. Low n-6/n-3 PUFA ratio improves lipid metabolism, inflammation, oxidative stress and endothelial function in rats using plant oils as n-3 fatty acid source. Lipids. 2016;51:49–59. 10.1007/s11745-015-4091-z. 26526061

[44] Liu HQ, Qiu Y, Mu Y, Zhang XJ, Liu L, Hou XH, et al. A high ratio of dietary n-3/n-6 polyunsaturated fatty acids improves obesity-linked inflammation and insulin resistance through suppressing activation of TLR4 in SD rats. Nutr Res. 2013;33:849–58. 10.1016/j.nutres.2013.07.004. 24074743

[45] Breslow JL. N-3 fatty acids and cardiovascular disease. Am J Clin Nutr. 2006;83:1477S-1482S. 10.1093/ajcn/83.6.1477S. 16841857

[46] Hagve TA, Lie Ø, Grønn M. The effect of dietary n-3 fatty acids on osmotic fragility and membrane fluidity of human erythrocytes. Scand J Clin Lab Invest. 1993;53:75–84. 10.3109/00365519309090699. 8327853

[47] An W, Dong X, Tan B, Yang Q, Chi S, Zhang S, et al. Dietary n-3 long chain polyunsaturated fatty acids affect the serum biochemical parameters, lipid-metabolism-related of gene expression and intestinal health of juvenile hybrid grouper (♀ *Epinephelus fuscoguttatu*s × ♂ *Epinephelus lanceolatus*). Aquac Nutr. 2021;27:1383–95. 10.1111/anu.13276.

[48] Bazan NG. Omega-3 fatty acids, pro-inflammatory signaling and neuroprotection. Curr Opin Clin Nutr Metab Care. 2007;10:136. 10.1097/MCO.0b013e32802b7030. 17285000

[49] Wei Y, Meng Y, Li N, Wang Q, Chen L. The effects of low-ratio n-6/n-3 PUFA on biomarkers of inflammation: a systematic review and meta-analysis. Food Funct. 2021;12:30–40. 10.1039/D0FO01976C. 33232407

[50] Li K, Huang T, Zheng J, Wu K, Li D. Effect of marine-derived n-3 polyunsaturated fatty acids on C-reactive protein, interleukin 6 and tumor necrosis factor α: a meta-analysis. PLoS ONE. 2014;9:e88103. 10.1371/journal.pone.0088103. PMC3914936 24505395

[51] Li Z, Zhao X, Jian L, Wang B, Luo H. Rumen microbial-driven metabolite from grazing lambs potentially regulates body fatty acid metabolism by lipid-related genes in liver. J Anim Sci Biotechnol. 2023;14:39. 10.1186/s40104-022-00823-y. PMC9990365 36879349

[52] Tedeschi LO, Muir JP, Naumann HD, Norris AB, Ramírez-Restrepo CA, Mertens-Talcott SU. Nutritional aspects of ecologically relevant phytochemicals in ruminant production. Front Vet Sci. 2021;8:628445. 10.3389/fvets.2021.628445. PMC7973208 33748210

[53] Magan JB, O’Callaghan TF, Kelly AL, McCarthy NA. Compositional and functional properties of milk and dairy products derived from cows fed pasture or concentrate-based diets. Compr Rev Food Sci Food Saf. 2021;20:2769–800. 10.1111/1541-4337.12751. 33949109

[54] Pacifici GM, Mogavero S, Giulian L, Rane A. Conjugation of benzoic acid with glycine in the human fetal and adult liver and kidney. Dev Pharmacol Ther. 1991;17:52–62. 10.1159/000457499. 1811921

[55] Chao C-T, Lin S-H. Uremic toxins and frailty in patients with chronic kidney disease: a molecular insight. Int J Mol Sci. 2021;22:6270. 10.3390/ijms22126270. PMC8230495 34200937

[56] Pallister T, Jackson MA, Martin TC, Zierer J, Jennings A, Mohney RP, et al. Hippurate as a metabolomic marker of gut microbiome diversity: modulation by diet and relationship to metabolic syndrome. Sci Rep. 2017;7:13670. 10.1038/s41598-017-13722-4. PMC5651863 29057986

[57] Sun H, Wang B, Wang J, Liu H, Liu J. Biomarker and pathway analyses of urine metabolomics in dairy cows when corn stover replaces alfalfa hay. J Anim Sci Biotechnol. 2016;7:49. 10.1186/s40104-016-0107-7. PMC5006375 27583137

[58] Su R, Zhou X, Steffen LM, Chen C. Identifying the ratio of circulating cinnamoylglycine to isoleucine as a biomarker linking diet quality, microbial metabolism, and type 2 diabetes risk in the Coronary Artery Risk Development in Young Adults (CARDIA) cohorts. J Nutr. 2026;156:101260. 10.1016/j.tjnut.2025.101260. PMC12974603 41371549

[59] Ramos SC, Jeong CD, Mamuad LL, Kim SH, Kang SH, Kim ET, et al. Diet transition from high-forage to high-concentrate alters rumen bacterial community composition, epithelial transcriptomes and ruminal fermentation parameters in dairy cows. Animals (Basel). 2021;11:838. 10.3390/ani11030838. PMC8002347 33809588

[60] Menni C, Zhu J, Le Roy CI, Mompeo O, Young K, Rebholz CM, et al. Serum metabolites reflecting gut microbiome alpha diversity predict type 2 diabetes. Gut Microbes. 2020;11:1632–42. 10.1080/19490976.2020.1778261. PMC7524143 32576065

[61] Manessis G, Kalogianni AI, Lazou T, Moschovas M, Bossis I, Gelasakis AI. Plant-derived natural antioxidants in meat and meat products. Antioxidants (Basel). 2020;9:1215. 10.3390/antiox9121215. PMC7761563 33276503

[62] Bernardi DM, Bertol TM, Pflanzer SB, Sgarbieri VC, Pollonio MAR. ω-3 in meat products: benefits and effects on lipid oxidative stability. J Sci Food Agric. 2016;96:2620–34. 10.1002/jsfa.7559. 26676414

[63] Laursen MF, Sakanaka M, von Burg N, Mörbe U, Andersen D, Moll JM, et al. Bifidobacterium species associated with breastfeeding produce aromatic lactic acids in the infant gut. Nat Microbiol. 2021;6:1367–82. 10.1038/s41564-021-00970-4. PMC8556157 34675385

[64] Dao Y, Zhang K, Lu X, Lu Z, Liu C, Liu M, et al. Role of glucose and 2-oxoglutarate/malate translocator (OMT1) in the production of phenyllactic acid and p-hydroxyphenyllactic acid, two food-borne pathogen inhibitors. J Agric Food Chem. 2019;67:5820–6. 10.1021/acs.jafc.9b01444. 31060357

[65] Rosa HJD, Rego OA, Silva CCG, Alves SP, Alfaia CMM, Prates JAM, et al. Effect of corn supplementation of grass finishing of Holstein bulls on fatty acid composition of meat lipids. J Anim Sci. 2014;92:3701–14. 10.2527/jas.2013-6982. 24987081

[66] Silva CCG, Domingos-Lopes MFP, Coelho MC, Rego OA, Rosa HJD. The effect of corn supplementation on α-tocopherol concentration and oxidative stability in three muscles of grass-fed Holstein bulls. Anim Prod Sci. 2022;63:96–100. 10.1071/AN22270.

[67] Shaw RA. Social organization and decision making in North American bison: implications for management [PhD thesis]. Logan, UT: Utah State University; 2012. 10.26076/9ba2-4d98.

[68] Osorio MT, Moloney AP, Brennan L, Monahan FJ. Authentication of beef production systems using a metabolomic-based approach. Animal. 2012;6:167–72. 10.1017/S1751731111001418. 22436164

[69] Zhang R, Jia W. Authenticity and traceability of goat milk: molecular mechanism of β-carotene biotransformation and accessibility. Food Chem. 2022;388:133073. 10.1016/j.foodchem.2022.133073. 35483296

[70] Fleming A, Provenza FD, Leroy F, van Vliet S, Elliot C, Hamlin M, et al. Connecting plant, animal, and human health using untargeted metabolomics. J Anim Sci. 2025;103:skaf254. 10.1093/jas/skaf254. PMC12451682 40831047

[71] Djuricic I, Calder PC. N-3 fatty acids (EPA and DHA) and cardiovascular health – updated review of mechanisms and clinical outcomes. Curr Atheroscler Rep. 2025;27:116. 10.1007/s11883-025-01363-2. PMC12628397 41247613

[72] Adhikari K, Chambers E IV, Miller R, Vázquez-Araújo L, Bhumiratana N, Philip C. Development of a lexicon for beef flavor in intact muscle. J Sens Stud. 2011;26:413–20. 10.1111/j.1745-459X.2011.00356.x.

[73] Vasta V, Priolo A. Ruminant fat volatiles as affected by diet. A review. Meat Sci. 2006;73:218–28. 10.1016/j.meatsci.2005.11.017. 22062292

[74] Fruet APB, Trombetta F, Stefanello FS, Speroni CS, Donadel JZ, De Souza ANM, et al. Effects of feeding legume-grass pasture and different concentrate levels on fatty acid profile, volatile compounds, and off-flavor of the M. longissimus thoracis. Meat Sci. 2018;140:112–8. 10.1016/j.meatsci.2018.03.008. 29549844

[75] O’Quinn TG, Legako JF, Woerner DR, Kerth CR, Nair MN, Brooks JC, et al. A current review of U.S. beef flavor II: managing beef flavor. Meat Sci. 2024;209:109403. 10.1016/j.meatsci.2023.109403. 38070358

[76] Ratajczak Z, Collins SL, Blair JM, Koerner SE, Louthan AM, Smith MD, et al. Reintroducing bison results in long-running and resilient increases in grassland diversity. Proc Natl Acad Sci U S A. 2022;119:e2210433119. 10.1073/pnas.2210433119. PMC9457053 36037376

[77] Spohn M, Bagchi S, Biederman LA, Borer ET, Bråthen KA, Bugalho MN, et al. The positive effect of plant diversity on soil carbon depends on climate. Nat Commun. 2023;14:6624. 10.1038/s41467-023-42340-0. PMC10587103 37857640

[78] Xue H, Mainville D, You W, Nayga RM. Consumer preferences and willingness to pay for grass-fed beef: empirical evidence from in-store experiments. Food Qual Prefer. 2010;21:857–66. 10.1016/j.foodqual.2010.05.004.

[79] United States Department of Agriculture, Food Safety and Inspection Service. Humane handling. https://www.fsis.usda.gov/inspection/compliance-guidance/humane-handling. Accessed 17 Jul 2026. 10.1093/tas/txac153 PMC9838095 36655233

