# Supplementary material for: Range-based finishing enhances omega-3 fatty acids and forage-derived metabolites in bison meat across a seven-treatment comparison
Source: J Anim Sci Biotechnol. 2026 Nov 5;17:200. doi: 10.1186/s40104-026-01513-9 (PMC13737706; doi:10.1186/s40104-026-01513-9)
Supplement: Supplementary file 1 [file 40104_2026_1513_MOESM1_ESM.docx]

**Supplemental Material**

**Table S1**. Fatty acid composition of bison *M. longissimus lumborum* across seven finishing systems. Values are mean ± SEM (% of total identified fatty acids); *n* = 16 animals per treatment (*n* = 15 for C17:0 in Pen-TMR-LOW, where one value was not recorded). *P* values indicate the overall treatment effect (one-way ANOVA); within a row, means that do not share a common superscript letter differ (Tukey's multiple comparisons test, *P* < 0.05). Fatty acids also reported in Table 3 of the main text are repeated here so that the full profile can be read in one place. Treatments: Pen-FREE-LOW = free-choice alfalfa hay, meadow hay and corn at low stocking density; Pen-TMR-LOW = bison total mixed ration (TMR) at low stocking density; Pen-TMR-HIGH = the same TMR at high stocking density; Pen-TMR-TRAD = traditional TMR at high stocking density (feedlot); Range-DIV = diverse native range; Range-LIM = low-diversity range; Range-LIM-CORN = low-diversity range plus corn. AA = arachidonic acid; ALA = α-linolenic acid; CLA = conjugated linoleic acid; DHA = docosahexaenoic acid; DPA = docosapentaenoic acid; EPA = eicosapentaenoic acid; LA = linoleic acid.

| **Fatty acid** | **Pen-FREE-LOW** | **Pen-TMR-LOW** | **Pen-TMR-HIGH** | **Pen-TMR-TRAD** | **Range-DIV** | **Range-LIM** | **Range-LIM-CORN** | ***P* value** |
| --- | --- | --- | --- | --- | --- | --- | --- | --- |
| ***Saturated fatty acids*** |  |  |  |  |  |  |  |  |
| C10:0 (capric acid) | 0.11 ± 0.01^b^ | 0.12 ± 0.01^b^ | 0.14 ± 0.01^b^ | 0.11 ± 0.01^b^ | 0.21 ± 0.01^a^ | 0.20 ± 0.01^a^ | 0.12 ± 0.01^b^ | < 0.0001 |
| C11:0 (undecylic acid) | 0.006 ± 0.000^a^ | 0.007 ± 0.000^a^ | 0.007 ± 0.000^a^ | 0.008 ± 0.000^a^ | 0.009 ± 0.002^a^ | 0.008 ± 0.001^a^ | 0.007 ± 0.000^a^ | 0.131 |
| C12:0 (lauric acid) | 0.008 ± 0.001^b^ | 0.008 ± 0.001^b^ | 0.009 ± 0.001^b^ | 0.009 ± 0.002^b^ | 0.016 ± 0.001^a^ | 0.014 ± 0.001^a^ | 0.009 ± 0.001^b^ | < 0.0001 |
| C14:0 (myristic acid) | 1.23 ± 0.04^a^ | 1.17 ± 0.05^ab^ | 1.16 ± 0.06^ab^ | 1.29 ± 0.05^a^ | 0.92 ± 0.05^c^ | 0.96 ± 0.04^bc^ | 1.19 ± 0.06^a^ | < 0.0001 |
| C15:0 (pentadecanoic acid) | 1.01 ± 0.26^a^ | 1.39 ± 0.39^a^ | 1.36 ± 0.34^a^ | 1.13 ± 0.28^a^ | 2.04 ± 0.55^a^ | 1.93 ± 0.47^a^ | 1.33 ± 0.34^a^ | 0.433 |
| C16:0 (palmitic acid) | 19.84 ± 0.29^a^ | 19.31 ± 0.33^ab^ | 19.20 ± 0.40^ab^ | 20.62 ± 0.40^a^ | 16.78 ± 0.39^cd^ | 16.35 ± 0.27^d^ | 18.26 ± 0.36^bc^ | < 0.0001 |
| C17:0 (heptadecanoic acid) | 1.13 ± 0.06^abc^ | 1.07 ± 0.04^bc^ | 1.03 ± 0.04^c^ | 1.01 ± 0.03^c^ | 1.28 ± 0.04^a^ | 1.21 ± 0.04^ab^ | 1.00 ± 0.03^c^ | < 0.0001 |
| C18:0 (stearic acid) | 13.79 ± 0.24^c^ | 14.42 ± 0.35^bc^ | 14.33 ± 0.27^c^ | 13.70 ± 0.24^c^ | 14.89 ± 0.18^bc^ | 15.67 ± 0.36^ab^ | 16.73 ± 0.42^a^ | < 0.0001 |
| C20:0 (arachidic acid) | 0.14 ± 0.01^b^ | 0.16 ± 0.01^ab^ | 0.16 ± 0.01^ab^ | 0.13 ± 0.01^b^ | 0.17 ± 0.01^ab^ | 0.19 ± 0.01^a^ | 0.15 ± 0.01^ab^ | < 0.01 |
| C22:0 (behenic acid) | 0.025 ± 0.002^c^ | 0.028 ± 0.003^bc^ | 0.030 ± 0.004^bc^ | 0.021 ± 0.002^c^ | 0.047 ± 0.007^a^ | 0.042 ± 0.004^ab^ | 0.025 ± 0.003^c^ | < 0.0001 |
| ***Monounsaturated fatty acids*** |  |  |  |  |  |  |  |  |
| C14:1n-5 (myristoleic acid) | 1.12 ± 0.27^a^ | 1.06 ± 0.28^a^ | 1.31 ± 0.35^a^ | 0.88 ± 0.21^a^ | 2.58 ± 0.64^a^ | 2.31 ± 0.60^a^ | 1.05 ± 0.29^a^ | 0.015 |
| C16:1n-7t (*trans*-palmitoleic acid) | 0.48 ± 0.18^a^ | 0.60 ± 0.20^a^ | 0.52 ± 0.20^a^ | 0.53 ± 0.20^a^ | 0.54 ± 0.13^a^ | 0.66 ± 0.18^a^ | 0.71 ± 0.22^a^ | 0.979 |
| C16:1n-7c (*cis*-palmitoleic acid) | 1.27 ± 0.04^a^ | 1.20 ± 0.05^a^ | 1.11 ± 0.06^a^ | 1.22 ± 0.03^a^ | 1.16 ± 0.05^a^ | 1.23 ± 0.04^a^ | 1.18 ± 0.03^a^ | 0.210 |
| C18:1n-9t (elaidic acid) | 0.32 ± 0.04^b^ | 0.31 ± 0.02^b^ | 0.40 ± 0.06^ab^ | 0.28 ± 0.01^b^ | 0.54 ± 0.06^a^ | 0.57 ± 0.06^a^ | 0.33 ± 0.02^b^ | < 0.0001 |
| C18:1n-9 (oleic acid) | 44.82 ± 0.63^a^ | 44.54 ± 0.96^a^ | 42.49 ± 0.75^a^ | 43.93 ± 0.52^a^ | 33.82 ± 0.94^b^ | 32.90 ± 0.70^b^ | 42.98 ± 0.90^a^ | < 0.0001 |
| C18:1n-7 (*cis*-vaccenic acid) | 2.93 ± 0.22^a^ | 2.31 ± 0.28^a^ | 2.65 ± 0.32^a^ | 3.07 ± 0.28^a^ | 2.91 ± 0.19^a^ | 2.76 ± 0.26^a^ | 2.25 ± 0.27^a^ | 0.212 |
| C20:1n-9 (*cis*-11-eicosenoic acid) | 0.17 ± 0.04^ab^ | 0.15 ± 0.03^b^ | 0.21 ± 0.04^ab^ | 0.15 ± 0.02^b^ | 0.31 ± 0.03^a^ | 0.31 ± 0.03^a^ | 0.21 ± 0.04^ab^ | < 0.01 |
| C24:1n-9 (nervonic acid) | 0.07 ± 0.01^ab^ | 0.08 ± 0.01^ab^ | 0.10 ± 0.01^ab^ | 0.09 ± 0.01^ab^ | 0.11 ± 0.01^a^ | 0.10 ± 0.01^a^ | 0.06 ± 0.01^b^ | < 0.01 |
| ***n-6 polyunsaturated fatty acids*** |  |  |  |  |  |  |  |  |
| C18:2n-6 (linoleic acid, LA) | 5.84 ± 0.31^b^ | 6.25 ± 0.48^b^ | 7.24 ± 0.49^b^ | 6.59 ± 0.33^b^ | 9.19 ± 0.56^a^ | 9.69 ± 0.43^a^ | 5.49 ± 0.41^b^ | < 0.0001 |
| C18:3n-6 (γ-linolenic acid) | 0.018 ± 0.003^b^ | 0.014 ± 0.003^b^ | 0.020 ± 0.004^b^ | 0.023 ± 0.011^b^ | 0.061 ± 0.010^a^ | 0.069 ± 0.012^a^ | 0.022 ± 0.002^b^ | < 0.0001 |
| C20:3n-6 (dihomo-γ-linolenic acid) | 2.46 ± 0.17^b^ | 2.61 ± 0.23^b^ | 3.03 ± 0.24^b^ | 2.42 ± 0.15^b^ | 4.48 ± 0.35^a^ | 4.32 ± 0.21^a^ | 2.42 ± 0.20^b^ | < 0.0001 |
| C20:4n-6 (arachidonic acid, AA) | 0.42 ± 0.03^bc^ | 0.44 ± 0.04^bc^ | 0.51 ± 0.04^bc^ | 0.25 ± 0.02^c^ | 1.48 ± 0.11^a^ | 1.54 ± 0.08^a^ | 0.60 ± 0.05^b^ | < 0.0001 |
| ***n-3 polyunsaturated fatty acids*** |  |  |  |  |  |  |  |  |
| C18:3n-3 (α-linolenic acid, ALA) | 0.56 ± 0.03^de^ | 0.58 ± 0.03^de^ | 0.71 ± 0.03^cd^ | 0.37 ± 0.04^e^ | 2.58 ± 0.10^b^ | 2.87 ± 0.11^a^ | 0.88 ± 0.06^c^ | < 0.0001 |
| C20:5n-3 (EPA) | 0.22 ± 0.01^abc^ | 0.23 ± 0.02^ab^ | 0.26 ± 0.02^a^ | 0.26 ± 0.02^a^ | 0.21 ± 0.01^abc^ | 0.19 ± 0.01^bc^ | 0.17 ± 0.01^c^ | < 0.0001 |
| C22:5n-3 (DPA) | 0.74 ± 0.06^bc^ | 0.77 ± 0.05^bc^ | 0.91 ± 0.06^b^ | 0.53 ± 0.03^c^ | 1.97 ± 0.13^a^ | 1.97 ± 0.09^a^ | 0.99 ± 0.08^b^ | < 0.0001 |
| C22:6n-3 (DHA) | 0.18 ± 0.02^cd^ | 0.20 ± 0.03^bcd^ | 0.24 ± 0.02^bc^ | 0.13 ± 0.01^d^ | 0.45 ± 0.04^a^ | 0.46 ± 0.03^a^ | 0.30 ± 0.03^b^ | < 0.0001 |
| ***Ruminant trans and conjugated fatty acids*** |  |  |  |  |  |  |  |  |
| C18:1t-11 (*trans*-vaccenic acid) | 0.80 ± 0.07^cd^ | 0.73 ± 0.10^d^ | 0.65 ± 0.07^d^ | 0.93 ± 0.09^bcd^ | 1.12 ± 0.08^abc^ | 1.35 ± 0.10^a^ | 1.24 ± 0.11^ab^ | < 0.0001 |
| *cis*-9, *trans*-11 CLA (rumenic acid) | 0.29 ± 0.05^a^ | 0.27 ± 0.04^ab^ | 0.20 ± 0.04^ab^ | 0.33 ± 0.04^a^ | 0.12 ± 0.03^b^ | 0.12 ± 0.03^b^ | 0.29 ± 0.05^ab^ | < 0.001 |
| ***Totals and ratios*** |  |  |  |  |  |  |  |  |
| Total saturated | 37.30 ± 0.37^a^ | 37.60 ± 0.52^a^ | 37.43 ± 0.65^a^ | 38.03 ± 0.57^a^ | 36.37 ± 0.75^a^ | 36.58 ± 0.80^a^ | 38.82 ± 0.71^a^ | 0.127 |
| Total monounsaturated | 51.01 ± 0.54^a^ | 50.10 ± 1.00^a^ | 48.58 ± 0.60^a^ | 50.00 ± 0.66^a^ | 41.66 ± 0.98^b^ | 40.53 ± 0.71^b^ | 48.56 ± 0.83^a^ | < 0.0001 |
| Total n-6 | 8.74 ± 0.51^b^ | 9.31 ± 0.74^b^ | 10.80 ± 0.77^b^ | 9.29 ± 0.47^b^ | 15.20 ± 1.01^a^ | 15.62 ± 0.71^a^ | 8.53 ± 0.65^b^ | < 0.0001 |
| Total n-3 | 1.69 ± 0.11^bc^ | 1.79 ± 0.12^bc^ | 2.13 ± 0.13^b^ | 1.28 ± 0.04^c^ | 5.22 ± 0.27^a^ | 5.49 ± 0.21^a^ | 2.34 ± 0.16^b^ | < 0.0001 |
| n-6:n-3 ratio | 5.20 ± 0.09^b^ | 5.15 ± 0.12^b^ | 5.03 ± 0.11^b^ | 7.25 ± 0.30^a^ | 2.88 ± 0.06^d^ | 2.83 ± 0.05^d^ | 3.61 ± 0.07^c^ | < 0.0001 |
| AA:EPA ratio | 1.92 ± 0.10^d^ | 1.89 ± 0.08^d^ | 1.98 ± 0.11^d^ | 1.00 ± 0.06^e^ | 6.94 ± 0.32^b^ | 8.17 ± 0.32^a^ | 3.63 ± 0.19^c^ | < 0.0001 |

**Table S2**. Targeted metabolite concentrations in bison *M. longissimus lumborum* (A) and in the feeds and forages offered in each finishing system (B), determined by LC–MS/MS. Meat values are mean ± SEM (µg/100 g wet weight); *n* = 13–18 animals per treatment. *P* values indicate the overall treatment effect (one-way ANOVA on detected values); ND = not detected; only compounds detected in at least one sample are shown. The Detected column gives the number of the 108 meat samples in which the compound was quantifiable; an em dash in the P value column marks compounds detected too sparsely for a treatment test (22 compounds, each quantifiable in 8 or fewer samples and in no more than two treatments). In panel (B), the final row gives the sum of all quantified compounds in each column, expressed as mg/100 g dry weight. For metabolites detected in only one sample within a treatment, the concentration is shown without an SEM. Feed and forage values are concentrations (µg/100 g dry weight, free + bound fractions combined) of the material offered in each treatment; Pen-FREE-LOW and Range-LIM-CORN are treatment-level composites of the offered ingredients rather than single consumed rations, and, because Pen-TMR-LOW and Pen-TMR-HIGH received the same total mixed ration, a single feed value (Pen-TMR) is shown for both pens in panel (B). Entries named "Unidentified isomer" are chromatographically resolved peaks that share the multiple-reaction-monitoring transitions of the compound given in parentheses but eluted at a different retention time and were not confirmed against an authentic standard; they are therefore reported without a compound assignment, and are retained in the column totals. Treatments: Pen-FREE-LOW = free-choice alfalfa hay, meadow hay and corn at low stocking density; Pen-TMR-LOW = bison total mixed ration (TMR) at low stocking density; Pen-TMR-HIGH = the same TMR at high stocking density; Pen-TMR-TRAD = traditional TMR at high stocking density (feedlot); Range-DIV = diverse native range; Range-LIM = low-diversity range; Range-LIM-CORN = low-diversity range plus corn.

**(A) Meat (*M. longissimus lumborum*)**

| **Metabolite** | **Detected (n/108)** | **Pen-FREE-LOW** | **Pen-TMR-LOW** | **Pen-TMR-HIGH** | **Pen-TMR-TRAD** | **Range-DIV** | **Range-LIM** | **Range-LIM-CORN** | ***P* value** |
| --- | --- | --- | --- | --- | --- | --- | --- | --- | --- |
| L-Kynurenine | 108 | 4.82 ± 0.61 | 3.82 ± 0.39 | 5.58 ± 0.73 | 5.34 ± 0.72 | 4.13 ± 0.51 | 4.11 ± 0.44 | 3.69 ± 0.60 | 0.152 |
| 3-(4-Hydroxyphenyl)lactic Acid | 108 | 55.58 ± 4.28 | 50.23 ± 4.30 | 50.42 ± 4.18 | 46.81 ± 4.31 | 38.00 ± 3.65 | 36.39 ± 3.47 | 43.52 ± 4.74 | 0.013 |
| catechol | 108 | 3.08 ± 0.40 | 3.12 ± 0.65 | 3.08 ± 0.32 | 6.83 ± 0.95 | 16.25 ± 2.12 | 18.34 ± 2.47 | 6.81 ± 0.61 | < 0.0001 |
| Shikimic acid | 108 | 6.65 ± 1.13 | 5.46 ± 0.68 | 6.71 ± 0.80 | 5.36 ± 0.63 | 14.51 ± 1.77 | 12.63 ± 1.49 | 6.27 ± 0.66 | < 0.0001 |
| 5-Feruloylquinic acid | 106 | 0.25 ± 0.14 | 1.86 ± 1.80 | 0.14 ± 0.06 | 0.11 ± 0.03 | 0.068 ± 0.014 | 0.28 ± 0.11 | 0.24 ± 0.07 | 0.454 |
| 2-Hydroxyisocaproic Acid | 108 | 13.38 ± 1.03 | 12.54 ± 0.93 | 13.13 ± 1.09 | 11.74 ± 0.98 | 15.33 ± 1.78 | 16.34 ± 1.26 | 12.54 ± 0.94 | 0.061 |
| 4-Hydroxybenzaldehyde | 108 | 0.81 ± 0.05 | 0.60 ± 0.04 | 0.72 ± 0.08 | 0.57 ± 0.04 | 0.75 ± 0.07 | 2.09 ± 1.39 | 0.72 ± 0.09 | 0.539 |
| Indoxyl Sulfate | 108 | 4.89 ± 0.56 | 4.32 ± 0.38 | 5.19 ± 0.52 | 3.81 ± 0.38 | 5.01 ± 0.53 | 5.04 ± 0.44 | 3.54 ± 0.45 | 0.069 |
| 3-Phenyllactic acid | 108 | 7.67 ± 0.59 | 7.91 ± 0.68 | 8.09 ± 0.70 | 7.80 ± 0.77 | 7.27 ± 0.92 | 5.81 ± 0.61 | 6.82 ± 0.77 | 0.287 |
| *trans*-Cinnamic acid | 108 | 53.28 ± 6.76 | 41.24 ± 6.28 | 44.13 ± 6.47 | 56.73 ± 7.99 | 56.63 ± 5.61 | 63.30 ± 17.41 | 49.08 ± 6.34 | 0.709 |
| p-Coumaric acid | 107 | 0.16 ± 0.04 | 0.23 ± 0.10 | 0.12 ± 0.02 | 0.11 ± 0.01 | 0.14 ± 0.03 | 1.15 ± 1.02 | 0.14 ± 0.01 | 0.576 |
| Salicylic acid | 108 | 1.53 ± 0.16 | 1.48 ± 0.20 | 1.79 ± 0.25 | 1.30 ± 0.14 | 1.67 ± 0.26 | 1.30 ± 0.12 | 1.43 ± 0.15 | 0.442 |
| *trans* Ferulic acid | 94 | 0.22 ± 0.07 | 0.39 ± 0.21 | 0.26 ± 0.08 | 0.16 ± 0.04 | 0.17 ± 0.05 | 0.48 ± 0.38 | 0.18 ± 0.03 | 0.838 |
| Quercetin 3-O-robinobioside | 108 | 1.66 ± 0.29 | 2.03 ± 0.56 | 2.16 ± 0.38 | 1.79 ± 0.26 | 2.85 ± 0.22 | 3.10 ± 0.37 | 2.48 ± 0.54 | 0.067 |
| Hyperoside | 24 | 2.48 ± 1.56 | 0.42 ± 0.34 | 1.09 ± 0.85 | 0.80 ± 0.24 | 0.39 | 0.22 | 1.86 | 0.744 |
| Cinnamoylglycine | 108 | 0.16 ± 0.04 | 0.13 ± 0.03 | 0.19 ± 0.03 | 0.33 ± 0.07 | 0.43 ± 0.07 | 0.37 ± 0.06 | 0.15 ± 0.02 | < 0.0001 |
| Hesperidin | 108 | 1.70 ± 1.07 | 0.57 ± 0.37 | 0.29 ± 0.09 | 0.84 ± 0.32 | 0.25 ± 0.04 | 0.77 ± 0.54 | 0.80 ± 0.36 | 0.517 |
| Diosmetin | 108 | 0.017 ± 0.002 | 0.075 ± 0.059 | 0.052 ± 0.017 | 0.019 ± 0.006 | 0.10 ± 0.05 | 0.053 ± 0.032 | 0.018 ± 0.005 | 0.345 |
| Chrysoeriol | 108 | 0.019 ± 0.002 | 0.078 ± 0.060 | 0.061 ± 0.021 | 0.019 ± 0.006 | 0.11 ± 0.05 | 0.058 ± 0.035 | 0.020 ± 0.006 | 0.357 |
| Hippuric acid | 108 | 22.75 ± 2.83 | 16.08 ± 2.00 | 16.65 ± 2.20 | 26.61 ± 2.33 | 73.51 ± 9.05 | 74.61 ± 8.38 | 30.31 ± 3.60 | < 0.0001 |
| p-Cresol sulfate | 108 | 92.30 ± 9.26 | 73.07 ± 7.58 | 93.19 ± 6.59 | 60.18 ± 5.40 | 85.45 ± 8.09 | 91.49 ± 7.90 | 75.84 ± 6.82 | 0.013 |
| p-Cresol Glucuronide | 108 | 12.50 ± 2.15 | 9.06 ± 1.22 | 9.01 ± 1.28 | 7.12 ± 0.78 | 10.10 ± 1.82 | 7.68 ± 0.69 | 9.28 ± 1.12 | 0.127 |
| 4-Caffeoylquinic acid | 7 | 3.93 | 5.87 | 3.04 | ND | ND | ND | 1.57 ± 0.17 | — |
| Trigonelline | 7 | ND | ND | 130.52 ± 17.10 | 3.19 | ND | ND | 7.05 | — |
| Hordenine | 5 | ND | ND | 119.14 ± 70.78 | ND | ND | ND | ND | — |
| Esculin | 4 | ND | ND | 7.48 ± 5.28 | ND | ND | ND | ND | — |
| Procyanidin B1 | 1 | ND | ND | 6.78 | ND | ND | ND | ND | — |
| 6,7-Dihydroxycoumarin | 4 | ND | ND | 4.88 ± 2.07 | ND | ND | ND | ND | — |
| Saponarin | 85 | 2.38 ± 0.53 | 1.36 ± 0.14 | 3.88 ± 2.12 | 1.42 ± 0.20 | 1.50 ± 0.11 | 1.61 ± 0.13 | 1.32 ± 0.10 | 0.001 |
| Glycitein | 1 | ND | ND | 52.18 | ND | ND | ND | ND | — |
| Glycitin | 1 | ND | ND | 5.82 | ND | ND | ND | ND | — |
| Orientin | 5 | ND | ND | 34.03 ± 23.18 | ND | ND | ND | ND | — |
| Vitexin-4-O-glucoside | 3 | ND | 0.46 | 19.64 | ND | ND | ND | ND | — |
| Morin | 8 | ND | 2.59 | 113.22 ± 72.61 | ND | ND | 3.08 | 16.65 | — |
| Vitexin | 4 | ND | ND | 99.20 ± 70.97 | ND | ND | ND | ND | — |
| Myrtillin | 49 | 0.36 ± 0.11 | 0.14 ± 0.03 | 4.25 ± 2.67 | 0.15 ± 0.02 | 0.14 ± 0.02 | 0.25 ± 0.05 | 1.11 ± 0.96 | 0.148 |
| Scopoletin | 6 | ND | 0.34 | 2.40 ± 2.04 | ND | ND | ND | ND | — |
| Kaempferol 7-O-glucoside | 7 | 6.11 | 4.33 | 45.83 ± 37.25 | ND | ND | ND | ND | — |
| Luteolin 7-O-Glucuronide | 5 | ND | 14.45 | 1566.74 ± 758.74 | ND | ND | ND | ND | — |
| Imperatorin | 4 | 8.21 | ND | 34.14 ± 7.70 | ND | ND | ND | ND | — |
| Homoplantaginin | 7 | 0.05 | 0.35 | 1.36 ± 0.54 | ND | ND | ND | ND | — |
| Naringenin Chalcone | 1 | ND | ND | 0.79 | ND | ND | ND | ND | — |
| Luteolin | 5 | ND | 0.54 | 19.46 ± 14.15 | ND | ND | ND | ND | — |
| Apigenin | 3 | ND | ND | 19.00 ± 11.87 | ND | ND | ND | ND | — |
| Isorhamnetin/3-Methylquercetin | 3 | ND | ND | 0.21 ± 0.13 | ND | ND | ND | ND | — |

**(B) Feeds and forages**

| **Metabolite** | **Pen-finished** | | | **Range-finished** | | |
| --- | --- | --- | --- | --- | --- | --- |
|  | **FREE-LOW** | **TMR** | **TMR-TRAD** | **DIV** | **LIM** | **LIM-CORN** |
| ***Hydroxybenzoic Acids & Phenolic Acids*** | | | | | | |
| 2,4-Dihydroxybenzoic acid | 364 | 351 | 176 | 89 | 1,218 | 62 |
| 2,6-dihydroxybenzoic acid | 671 | 1,032 | 1,889 | 150 | 1,188 | 296 |
| 2-Hydroxy-5-methoxybenzoic Acid | 1 | 2 | *ND* | *ND* | 5 | 4 |
| 2-Hydroxycinnamic acid | *ND* | *ND* | 7 | *ND* | 9 | *ND* |
| 3,4,5-Trimethoxybenzoic Acid | *ND* | 10 | *ND* | *ND* | *ND* | 128 |
| 3,4-Dicaffeoylquinic acid | 13 | 34 | 79 | 99 | 2,159 | 191 |
| 3,4-Dihydroxybenzaldehyde | 138 | 67 | 140 | 549 | 268 | 189 |
| 3,4-Dihydroxybenzoic acid | 134 | 265 | 434 | 627 | 326 | 95 |
| 3-(2-Hydroxyphenyl)propanoic acid | *ND* | *ND* | *ND* | *ND* | 82 | *ND* |
| 3-(4-Hydroxyphenyl)lactic Acid | 34 | 35 | 429 | 54 | 20 | 23 |
| 3-Caffeoylquinic acid | 647 | 490 | 1,015 | 6,755 | 25,723 | 10,598 |
| 3-Feruloylquinic acid | 4,927 | 1,542 | 957 | 12,986 | 20,153 | 11,585 |
| 3-Hydroxybenzoic acid | 404 | 345 | 591 | 660 | 555 | 217 |
| 3-Hydroxybenzyl Alcohol | *ND* | 24 | 81 | *ND* | 344 | 89 |
| 3-Hydroxyphenylacetic acid | 80 | 65 | 367 | 11 | 21 | 8 |
| 3-Phenyllactic acid | 62 | 97 | 5,242 | 40 | 114 | 47 |
| 4-Caffeoylquinic acid | 227 | 232 | 358 | 5,317 | 8,822 | 2,447 |
| 4-Hydroxybenzaldehyde | 6,642 | 1,744 | 2,279 | 11,632 | 7,331 | 5,881 |
| Unidentified isomer (4-hydroxybenzaldehyde transitions) | 8,505 | 2,178 | 2,830 | 14,785 | 8,776 | 7,348 |
| 4-Hydroxybenzoic acid | 317 | 232 | 440 | 542 | 437 | 170 |
| 4-Hydroxybenzyl Alcohol | 644 | 93 | 624 | 412 | 1,059 | 102 |
| 4-Hydroxyphenylacetic Acid | 85 | 76 | 403 | 21 | 22 | 10 |
| 4-Methylcatechol-1-sulfate | *ND* | *ND* | *ND* | *ND* | 16 | *ND* |
| 5-Feruloylquinic acid | 5,306 | 1,402 | 1,113 | 11,870 | 20,253 | 15,264 |
| Benzoic acid | 3,106 | 1,963 | 2,807 | 1,688 | 1,637 | 1,630 |
| Caffeic acid | 111 | 107 | 190 | 2,140 | 3,860 | 1,352 |
| catechol | 77 | 340 | 587 | 703 | 479 | 346 |
| Chlorogenic acid | 504 | 132 | 44 | 5,077 | 5,380 | 1,303 |
| Cinnamamide | 1 | 1 | 1 | 4 | 30 | 34 |
| Dihydroferulic acid 4-glucuronide | 474 | 52 | 385 | 1,659 | 2,478 | 1,463 |
| Ellagic acid | 214 | 103 | 367 | 1,635 | 214 | 118 |
| Hydroxytyrosol | 164 | 131 | 253 | 194 | 1,303 | 1,982 |
| Methyl caffeate | 2,311 | *ND* | *ND* | 1,948 | 14,825 | 13,059 |
| Orselinic acid | 3 | *ND* | *ND* | 11 | 6 | 117 |
| p-Coumaric acid | 10,331 | 15,820 | 23,450 | 3,239 | 5,047 | 8,419 |
| Pyrogallol-2-sulfate-1 | 101 | 40 | 3,170 | 473 | 421 | 26 |
| Resorcinol | 879 | 1,079 | 1,531 | 119 | 1,204 | 308 |
| Shikimic acid | 15 | 3 | 11 | 6 | 46 | 36 |
| Sinapic acid | 1,958 | 925 | 1,489 | 1,377 | 1,689 | 1,763 |
| Syringic acid | 1,323 | 728 | 1,537 | 5,910 | 2,624 | 2,392 |
| trans Ferulic acid | 19,679 | 17,624 | 24,289 | 22,416 | 17,542 | 20,223 |
| Vanillic acid | 4,095 | 2,364 | 3,262 | 10,386 | 6,284 | 4,741 |
| Vanillic acid 4-beta-D-glucopyranoside | 5,343 | 1,110 | 7,430 | 6,117 | 4,800 | 2,904 |
| ***Flavonoids*** | | | | | | |
| 6,7,4-Trihydroxyisoflavone | *ND* | *ND* | 1,834 | *ND* | *ND* | *ND* |
| 7,4-Dihydroxyflavone | 71 | 366 | 124 | 6 | 28 | 13 |
| Apigenin | 2,226 | 3,679 | 1,431 | 2,210 | 2,209 | 1,419 |
| Aromadendrin | 16 | 9 | 24 | 12 | 2 | 1 |
| Calycosin | 28 | 4 | 38 | 37 | 182 | 37 |
| Chrysoeriol | 726 | 729 | 570 | 1,870 | 2,926 | 1,462 |
| Coumestrol | 99 | 148 | *ND* | *ND* | *ND* | *ND* |
| Cyanidin | 14 | *ND* | 109 | *ND* | 1,770 | 313 |
| Daidzein | 24 | 121 | 6 | *ND* | 53 | 86 |
| Unidentified isomer (daidzein transitions) | 14,314 | 3,371 | 5,656 | *32,374* | 18,658 | 15,465 |
| Diosmetin | 702 | 738 | 585 | 1,751 | 3,040 | 1,410 |
| Eriodictyol 7-O-glucoside | 3 | 1 | 1 | 41 | 72 | 13 |
| Formononetin | 29 | 670 | 83 | 1 | 11 | 17 |
| Genkwanin | 10 | *ND* | 16 | 8 | 82 | *ND* |
| Hesperetin | 11 | *ND* | 52 | *ND* | *ND* | *ND* |
| Hesperetin 7-O-glucoside | 339 | 347 | 2,799 | 2,822 | 786 | 513 |
| Hesperidin | 2 | 5 | *ND* | *ND* | *ND* | 57 |
| Hispidol | 69 | 343 | 102 | 5 | 27 | 4 |
| Unidentified isomer (homoplantaginin transitions) | 29 | 2 | 2 | *42* | 314 | 211 |
| Hyperoside | 219 | 63 | 2,203 | 3,988 | 555 | 493 |
| Isorhamnetin-3-O-beta-D-Glucoside | 9 | *ND* | 24 | 16 | 22 | 12 |
| Isorhamnetin/3-Methylquercetin | 4 | 7 | 12 | 39 | 5 | 6 |
| Juglanin | 16 | *ND* | *ND* | 10 | 4 | 3 |
| Kaempferide | 337 | 834 | 575 | 524 | *ND* | *ND* |
| Kaempferitrin | *ND* | 11 | 11 | *ND* | *ND* | *ND* |
| Kaempferol | 184 | 120 | 156 | 97 | *ND* | *ND* |
| Kaempferol 3-glucuronide | 0 | 7 | 1,538 | 651 | *ND* | 1 |
| Kaempferol 7-O-glucoside | 267 | 92 | 391 | 1,169 | 10,736 | 909 |
| Kaempferol-3-glucoside | 924 | 137 | 451 | 163 | 41 | 71 |
| Keracyanin | 83,030 | 18,731 | 3,422 | 8,376 | 243,942 | 118,246 |
| Linarin | 4 | *ND* | 20 | 31 | 148 | 13 |
| Luteolin | 496 | 323 | 903 | 4,078 | 6,183 | 2,805 |
| Luteolin 7-glucoside | 346 | 91 | 38 | *ND* | *ND* | *ND* |
| Luteolin 7-O-Glucuronide | 4,074 | 6,201 | 33,816 | 21,118 | 48,807 | 13,699 |
| Luteolin 7-O-Rutinoside | 15 | 45 | 2,601 | 24 | 1,641 | *ND* |
| Mellein | 148 | 6 | 6 | 298 | 302 | 243 |
| Morin | 387 | 113 | 12,490 | 19,738 | 2,631 | 241 |
| Myricetin 3-galactoside | 58 | 63 | 129 | 236 | 151 | 118 |
| Naringenin | 150 | 92 | 68 | 755 | 356 | 463 |
| Naringin | *ND* | *ND* | *ND* | 29 | *ND* | *ND* |
| Neoeriocitrin | 29 | 27 | *ND* | 101 | 90 | 35 |
| Nicotiflorin | 88 | 41 | 2,390 | *ND* | 4,331 | *ND* |
| Ononetin | 44 | 70 | 12 | 6 | 61 | 24 |
| Orientin | 144 | 240 | 804 | 2,453 | 2,822 | 644 |
| Prunin | 14 | 33 | 362 | 65 | 53 | 35 |
| Quercetin | 127 | 375 | 565 | 634 | 248 | 370 |
| Quercetin 3-O-glucuronide | 57 | 450 | 4,541 | 8,069 | 247 | 58 |
| Quercetin 3-O-robinobioside | 58 | 102 | 8,835 | 3,923 | 942 | 177 |
| Saponarin | 651 | 89 | 181 | 802 | 13,330 | 11,782 |
| Schaftoside | 1,260 | 1,100 | 1,656 | 16,412 | 11,534 | 8,300 |
| Scutellarein | 18 | 24 | 23 | *ND* | *ND* | *ND* |
| Swertisin | 1,098 | 874 | 1,810 | 7,351 | 21,020 | 55,375 |
| Taxifolin | *ND* | *ND* | *ND* | 169 | 18 | 3 |
| Vitexin | 578 | 488 | 6,556 | 9,762 | 9,192 | 3,938 |
| Vitexin 2-O-rhamnoside | 387 | 33 | 245 | 549 | 1,486 | 390 |
| Vitexin-4-O-glucoside | 1,649 | 128 | 2,036 | 1,587 | 3,649 | 54,834 |
| ***Coumarins*** | | | | | | |
| 6,7-Dihydroxycoumarin | 96 | 251 | 202 | 1,147 | 206 | 94 |
| 7-hydroxy Coumarin sulfate | 0 | *ND* | *ND* | 4 | 17 | 11 |
| Esculin | 10 | 10 | 11 | 527 | 216 | 192 |
| Fraxin | 0 | 4 | 2 | 15 | 3 | 1 |
| Imperatorin | 4,199 | 5,716 | 2,680 | 2,035 | 2,232 | 1,843 |
| Scopoletin | 56 | 40 | 65 | 379 | 166 | 54 |
| ***Alkaloids*** | | | | | | |
| Hercynine | 161 | 28 | 49 | 340 | 185 | 111 |
| Hordenine | 736 | 418 | 97 | 351 | 535 | 494 |
| L-Kynurenine | 1 | 8 | 6 | 26 | 4 | 2 |
| Nuciferine | 1 | *ND* | 3 | *ND* | *ND* | *ND* |
| Stachydrine | 16,441 | 35,531 | 57,558 | 33,601 | 8,731 | 6,734 |
| Theobromine | 15 | 3 | *ND* | 14 | 8 | 13 |
| Theophylline | 2 | 1 | 4 | 3 | *ND* | 3 |
| Trigonelline | 2,014 | 1,217 | 3,675 | 746 | 874 | 831 |
| ***Lignans*** | | | | | | |
| Arctigenin | 137 | 4 | 8 | 461 | 226 | 204 |
| Matairesinol | 1 | 1 | 5 | 6 | 9 | 3 |
| Pinoresinol Diglucoside | 131 | 34 | 50 | 1,104 | 666 | 128 |
| Secoisolariciresinol | 83 | 48 | 47 | 177 | 43 | 29 |
| Sesamin | *ND* | *ND* | *ND* | 77 | 185 | *ND* |
| ***Stilbenes*** | | | | | | |
| Polydatin | 10 | 8 | 29 | 39 | 42 | 7 |
| Pterostilbene | 15 | *ND* | *ND* | *ND* | 2 | 13 |
| ***Other*** | | | | | | |
| 1-Salicylate Glucuronide | 5 | 115 | 5 | *ND* | 41 | 6 |
| 2 hydroxybenzoic acid | 3,925 | 2,500 | 2,871 | 279 | 707 | 390 |
| 2-Hydroxy-4-methoxybenzaldehyde | 452 | *ND* | *ND* | 280 | 538 | 714 |
| 2-Hydroxyisocaproic Acid | 13 | *ND* | 1,694 | 10 | 6 | 9 |
| 4-hydroxyhippuric acid | 1 | 2 | 2 | *ND* | 1 | 1 |
| 4-Methoxyphenyl-beta-D-Glucopyranoside | 89 | 73 | 69 | 199 | 100 | 131 |
| Chrysin | 1 | 1 | 1 | 7 | 5 | 1 |
| Cinnamoylglycine | 2 | 1 | 2 | *ND* | 3 | *ND* |
| Emodin 8-glucoside | *ND* | *ND* | 12 | *ND* | *ND* | *ND* |
| Gentisic acid | 76 | 1,518 | *ND* | 248 | 316 | *ND* |
| Indole-3-Carboxaldehyde | 364 | 234 | 234 | 304 | 181 | 221 |
| Indoxyl Sulfate | 2 | 1 | 5 | *ND* | 14 | *ND* |
| Ononin | 0 | 34 | 15 | *ND* | 2 | 7 |
| p-Cresol Glucuronide | 0 | *ND* | *ND* | *ND* | 5 | *ND* |
| p-Cresol sulfate | 2 | 1 | *ND* | *ND* | 16 | 1 |
| Puerarin | 1 | 23 | 18 | *ND* | 7 | *ND* |
| **Total quantified phenolics (mg/100 g)** | **224.5** | **142.2** | **262.0** | **328.5** | **599.8** | **423.5** |
